# The prognostic significance and potential mechanism of DBF4 zinc finger in hepatocellular carcinoma

**DOI:** 10.1038/s41598-024-60342-w

**Published:** 2024-05-09

**Authors:** Zhongkai Wu, Lilong Zhang, Xinyi Li, Li Liu, Tianrui Kuang, Zhendong Qiu, Wenhong Deng, Weixing Wang

**Affiliations:** 1 Department of General Surgery, Renmin Hospital of Wuhan University, No. 238, Jiefang Road, Wuchang District, Wuhan, 430060 Hubei Province China; 2 Hubei Key Laboratory of Digestive System Disease, No. 238, Jiefang Road, Wuchang District, Wuhan, 430060 Hubei Province China

**Keywords:** DBF4, ERBB2, Hepatocellular carcinoma, Prognosis, Proliferation, Migration, Invasion, Cancer, Cell biology

## Abstract

DBF4 zinc finger (DBF4) is a critical component involved in DNA replication and cell proliferation. It acts as a positive regulator of the cell division cycle 7 kinase. In this study, our investigation encompassed the impact of DBF4 on hepatocellular carcinoma (HCC) progression and delved into the potential mechanisms. We utilized open-access databases like TCGA and GEO to analyze the association between DBF4 and 33 different tumor types. We also conducted immunohistochemistry experiments to validate the expression of DBF4 in HCC, STAD, COAD, READ, PAAD, and LGG. Furthermore, we employed lentiviral transduction to knockdown DBF4 in HLF and SMMC cells, as well as to overexpress DBF4 in Huh7 cells. Subsequently, we evaluated the impact of DBF4 on proliferation, migration, and invasion of hepatocellular carcinoma cells. RNA sequencing and KEGG pathway enrichment analysis were also conducted to identify potential pathways, which were further validated through WB experiments. Finally, pathway inhibitor was utilized in rescue experiments to confirm whether DBF4 exerts its effects on tumor cells via the implicated pathway. Our findings revealed that DBF4 exhibited significant expression levels in nearly all examined tumors, which were further substantiated by the results of immunohistochemistry analysis. High DBF4 expression was correlated with poor overall survival (OS), disease-specific survival (DSS), progression-free interval (PFI), disease-free interval (DFI), relapse-free interval (RFI) in majority of tumor types, particularly in patients with HCC. In vitro experiments demonstrated that inhibition of DBF4 impaired the proliferative, migratory, and invasive abilities of HCC cells, whereas overexpression of DBF4 promoted these phenotypes. Sequencing results indicated that DBF4 may induce these changes through the ERBB signaling pathway. Further experimental validation revealed that DBF4 activates the ERBB signaling pathway, leading to alterations in the JNK/STAT, MAPK, and PI3K/AKT signaling pathways, thereby impacting the proliferative, migratory, and invasive abilities of tumor cells. Lastly, treatment of Huh7 cells overexpressing DBF4 with the ERBB2 inhibitor dacomitinib demonstrated the ability of ERBB2 inhibition to reverse the promoting effect of DBF4 overexpression on the proliferative, migratory, and invasive abilities of HCC cells. DBF4 plays a pivotal oncogenic role in HCC by promoting the ERBB signaling pathway and activating its downstream PI3K/AKT, JNK/STAT3, and MAPK signaling pathways. DBF4 may serve as a prognostic biomarker for patients with HCC.

## Introduction

DBF4 zinc finger (DBF4), also known as ASK, CHIF, DBF4A, and ZDBF1, functions as a positive regulatory subunit of the cell division cycle 7 (CDC7) kinase, playing a pivotal role in DNA replication and cell proliferation. The DBF4-CDC7 complex phosphorylates the Mcm2 helicase at Ser40 and Ser53, enabling the initiation of eukaryotic DNA replication^1^. CDC7 is expressed consistently throughout the cell cycle but only becomes active when bound to its regulatory subunit^2,3^. On the other hand, DBF4 is an unstable protein that reaches its highest abundance during the late G1 phase and undergoes degradation at the end of mitosis. Consequently, the kinase activity of CDC7 peaks at the onset of cellular DNA synthesis and diminishes at the end of mitosis^2,4,5^. Interestingly, apart from its role in activating CDC7, DBF4 also interacts with other crucial cell cycle kinases like Rad53^6,7^ and CDC5^8,9^, highlighting its critical regulatory function in cell cycle progression.

The oncogenic role of DBF4 in specific cancers is garnering increasing attention in research. Nambiar et al. conducted a study that confirmed the upregulation of DBF4 in melanomas, both at mRNA and protein levels, with higher levels of DBF4 being associated with lower relapse-free survival. Depletion of DBF4 using siRNA resulted in significant suppression of cell survival and proliferation in human melanoma cell lines^10^. Qi et al.^11^ discovered high expression of DBF4 in LUSC cells, and demonstrated that overexpression of DBF4 promotes proliferation, migration, invasion, and epithelial-mesenchymal transition in LUSC. Moreover, DBF4 has been implicated in tumor drug resistance. A study revealed a sixfold higher expression of DBF4 in docetaxel-resistant prostate cancer cells compared to docetaxel-sensitive cells^12^. Wang et al.^13^ reported that DBF4 enhances cell proliferation and migration and reduces sensitivity to 5-Fu in gastric cancer cell lines.

DBF4-mediated phosphorylation cascades involving integrins and collagens have been identified as critical regulators of cell adhesion and migration. Inhibition of DBF4 has been shown to suppress the migration and adhesion of prostate (PC3), breast (MB231), and cervical (HeLa) cancer cells, thereby impeding metastasis^14^. A recent study has reported a specific upregulation of DBF4 through the loss of DUSP4 and p53 in breast cancer, which contributes to tumorigenesis by mediating cell cycle checkpoint escape and promoting cell replication^15^. Similarly, in Ewing's sarcoma, Jeffrey et al.^16^ observed that inhibition of DBF4 leads to abnormal cell mitosis and ultimately results in mitotic catastrophe. Despite investigations suggesting that DBF4 can promote hepatocellular carcinoma (HCC) progression by activating STAT3^17^, considering the complex physiological functions of DBF4 and its potential impacts on various signaling pathways, the precise mechanisms through which DBF4 promotes HCC still warrant further investigation.

In this investigation, a bioinformatic analysis revealed a potential association between DBF4 and the development of HCC. Subsequently, an array of experimental techniques, including RNA sequencing, were employed to examine the impact of DBF4 on HCC progression and elucidate the molecular pathways through which DBF4 drives HCC advancement.

## Materials and methods

### Chemicals and antibodies

Acrylamide, bromophenol blue, 1.5 M Tris-HCL, 1.0 M Tris-HCL, sodium dodecyl sulphate (SDS) and tetramethylene diamine (TEMED) was purchased from Servicebio (Wuhan, China). RPMI-1640 and DMEM were purchased from Servicebio (Wuhan, China). Fetal bovine serum was purchased from QMSuero (Wuhan, China). Antibiotics was purchased from Biosharp (Hefei, China). Antibodies for DBF4 was purchased from Immunoway (Jiangsu, China). ERBB2 and p-ERBB2^Y1221/1222^ were purchased from Abclonal (Wuhan, China). STAT3 and p-STAT3 were purchased from Abmart (Shanghai, China). PI3K, p-PI3K, AKT and p-AKT were purchased from Cell Signaling Technology. P38 MAPK, p-P38 MAPK, N-CADHERIN, VIMENTIN and SNAIL were purchased from Proteintech (Wuhan, China). MMP9 and GAPDH were purchased from Servicebio. Dacomitinib (a specific, irreversible inhibitor of the ERBB family that targets EGFR, ERBB2, and ERBB4) were purchased from MedChemExpress (Shanghai, China). Lipo8000™ transfection reagent was purchased from FuShen (Shanghai, China). Kcockdown and overexpression plasmids for lentivirus production was synthesized by Miaoling Biology (Wuhan, China).

### Cell lines and culture conditions

293T, HLF and Huh7 were cultured in DMEM supplemented with 10% fetal bovine serum, and 1% antibiotics and SMMC-7721 were cultured in RPMI-1640 supplemented with 10% fetal bovine serum, and 1% antibiotics. For lentivirus production, pLV2-U6-DBF4(human)-shRNA2-PGK-EGFP-Puro (8 µg), psPAX2 (6 µg), and pMD2G (2 µg) were purchased from MiaoLing Plasmid Platform (Wuhan, China) and were cotransfected into 293T cells. At 48 h after transfection, the virus-containing supernatants were collected and added to ex-ponentially growing HLF and SMMC cells. DBF4 stable knockdown cells were achieved by 2-day puromycin (2 µg/mL, Cayman Chemical, USA) selection. The viral vectors overexpressing DBF4 (pLV3-CMV-DBF4(human)-3 × FLAG-EF1a-CopGFP-Puro) were generated using identical techniques and subsequently introduced into ex-ponentially growing Huh7 cells. Stable overexpression of DBF4 in cells was achieved through the use of a 2-day puromycin (2 µg/mL, Cayman Chemical, USA) selection process. 24 h prior to conducting some experimental procedures, a part of Huh7 cells stably overexpressing DBF4 were selected and cultured in a medium supplemented with dacomitinib (1 μmol/L). The utilized cell lines (293T, HLF, SMMC-7721 and Huh7) originated from the Experimental Laboratory of General Surgery at Hospital of Wuhan University.

### Data collection and expression analysis of DBF4

We obtained data from the Genotype-Tissue Expression (GTEx) and The Cancer Genome Atlas (TCGA) datasets using the Xena Browser. Additionally, we retrieved the GSE121248, GSE102079, GSE62232, GSE17856, GSE55092, GSE45267, GSE112790, and Liver Cancer-RIKEN, JP (LIRI-JP) datasets from the Gene Expression Omnibus (GEO) and the International Cancer Genome Consortium Data Portal (ICGC). For the TCGA data, we calculated the number of normal samples available per tumor type and selected tumors with at least five normal samples for comparison with cancer and paracancer tissues. To evaluate the disparities in DBF4 expression levels between tumor and non-tumor control samples, we performed an analysis using the Oncomine™^18^ database, applying the following thresholds: THRESHOLD (PVALUE) ≤ 0.05, THRESHOLD (FOLD CHANGE) = ALL, and THRESHOLD (GENE RANK) in the top 10% for all data types. Statistical comparisons between the two groups were conducted using the Wilcoxon test, while the Kruskal–Wallis test was employed for comparisons among three groups. To visualize the data, we generated box plots using the “ggpubr” and “ggplot2” R packages. Moreover, we utilized the Integrative Molecular Database of Hepatocellular Carcinoma (HCCDB)^19^ database to analyze changes in DBF4 expression specifically in hepatocellular carcinoma (HCC).

### Survival analysis and independent prognostic analysis

We analyzed the TCGA dataset to probe the association of DBF4 expression with OS, DSS, DFI, and PFI. This analysis Divide those above the median expression of DBF4 into high expression groups, and the rest into low expression groups. To assess the difference in survival between these two groups, we employed the “survival” and “survminer” R packages for Kaplan–Meier analysis. Furthermore, the Cox proportional hazards regression analysis was used to evaluate the hazard ratio, and a forest plot was generated using the “survival” and “forestplot” R packages. To explore the association of DBF4 expression with relapse-free survival (RFS) across 21 distinct types of cancer, we utilized the Kaplan–Meier Plotter, which incorporates data from GEO, European Genome-phenome Archive, and TCGA, with patients divided into high and low expression groups based on the median^20^. To validate the prognostic value of DBF4 in hepatocellular carcinoma (HCC), we referred to the HCCDB^19^ database (ICGC-LIRI-JP data) and the Long-term Outcome and Gene Expression Profiling Database of pan-cancers (LOGpc)^21^ database, with patients categorized based on the upper 50%. Univariate and multivariate Cox regression analyses were performed using the TCGA-HCC cohort data, and forest plots were constructed using the “forestplot” R package to display the P-value, hazard ratio (HR), and 95% confidence interval (CI) for each variable. Subsequently, using the results, we created a nomogram with the intention of precisely forecasting the probability of 1-, 2-, 3-, and 5-year OS.

### Cytotoxicity assay

Cell proliferative capacity is assessed using the CCK-8 assay. Cells (5 × 10^3^) are initially plated in 96-well plates and cultivated for 24 h, 48 h, or 72 h. Following these intervals, the culture medium is discarded, and each well is supplied with 100 μL of fresh medium, coupled with 10 μL of the CCK-8 solution (Dojido, Tokyo, Japan), and incubated for 2 h thereafter. Using an enzyme immunoassay (ZY057877, Beijing Zhongyi Technology Co., Ltd., Beijing, China) to measure the optical density at 450 nm. The mean values were calculated from six duplicate wells. The experiment was repeated three times.

### Wound healing assay

The capacity of cellular migration is assessed via the wound healing assay. Cells are allowed to achieve approximately 100% confluence in six-well plates, followed by the generation of a series of linear wounds using a sterile 200 μl pipette tip. Subsequent to the initiation of wounds, the system is subjected to a single PBS wash to discard dead or adherent cells found at the wound sites. The cultivation is executed in a serum-free medium, with images of the wound sites captured at 0 h and 24 h intervals. For each cell type, two replicates are undertaken, yielding eight possible fields of view for image capture. The experiment was repeated three times.

### Motility and invasion assay

To evaluate the invasion and migration of cells, 100 μL of cell (3 × 10^4^ for migration and 8 × 10^4^ for invasion) suspensions without FBS were added to the upper chamber of 24-well Transwell chambers coated or uncoated with 90 μL Matrigel (BD, USA), and 600 µL of DMEM containing 10% FBS was added to the lower chamber. After 48 h (for migration) or 60 h (for invasion), removed the upper chamber and fixed the cells on the underside of the membrane with 4% paraformaldehyde for 30 min, followed by staining with a 0.1% crystal violet solution for 20 min. Five random fields of view were selected and counted under a 200-fold inverted microscope.

### RNA sequencing

To explore the potential underlying mechanisms of the influence of DBF4 expression on cancer progression, we knocked down DBF4 in HLF cells and sent them to Biomarker for transcriptome sequencing and Gene Set Enrichment Analysis. To begin, total RNA was extracted from sh-NC and sh-DBF4 transfected HLF cells separately. The concentration, purity, and integrity of the RNA were assessed using a NanoDrop 2000 spectrophotometer and an Agilent 2100/LabChip GX system. Upon passing quality checks, mRNA from eukaryotic organisms was enriched using magnetic beads containing Oligo (dT) primers. The enriched mRNA was then fragmented using Fragmentation Buffer, and the fragmented mRNA served as a template for the synthesis of the first-strand cDNA and second-strand cDNA, followed by cDNA purification. The purified double-stranded cDNA underwent end repair, A-tailing, and adapter ligation, followed by size selection using AMPure XP beads. Finally, a cDNA library was obtained through PCR enrichment. After library construction, an initial quantification was performed using the Qubit 3.0 fluorometer, requiring a concentration of 1 ng/µL or higher. Subsequently, the insert size of the library fragments was assessed using the Qsep400 high-throughput analysis system. Once the expected insert size was confirmed, the effective concentration of the library (with a concentration of > 2 nM) was accurately quantified using qPCR to ensure library quality. After passing library quality control, paired-end (PE) sequencing in PE150 mode was conducted using the Illumina NovaSeq6000 platform. Following sequencing, the raw data were filtered to obtain clean data, which were then aligned to a reference genome to generate mapped data.

### Immunohistochemical staining

Tumor and paracancerous tissue samples were obtained from 40 cases of HCC, 10 cases of STAD, 10 cases of COAD, 10 cases of READ, 10 cases of PAAD, and 10 cases of LGG at Renmin Hospital of Wuhan University, China. On these tumor tissues, immunohistochemistry analysis was carried out. The paraffin sections were briefly incubated in an oven for 80 min at 70 °C. After xylene deparaffinization twice (20 min each), gradient ethanol hydration (5 min each). Antigen retrieval was achieved by placing the sections in a boiling sodium citrate buffer solution and cooking for an additional 4 min. Then, the slides were treated with a 3% hydrogen peroxide solution for 15 min. The sections were incubated overnight at 4 °C with the anti-DBF4 antibody (rabbit, immunoway, 1:100), then incubated with a second antibody. We employed 3,5-diaminobenzidine (DAB) to visualize the staining effects and each slide was then meticulously examined and captured blindly using a light microscope. Quantitative analysis of immunostained sections was performed using digital image analysis and the Image-Pro Plus 6.0 program (Media Cybernetics Inc, Bethesda, USA). The percentage of DBF4-positive nuclei was used to describe the immunostained sections. Three randomly selected photos from each segment were obtained to calculate the mean value for statistical comparison. The median value was used as the threshold to define high and low expression groups. The data were expressed as the mean and standard deviation. Statistical analysis was performed using SPSS 21.0 software (SPSS Inc., Chicago, USA).

### Western blotting

To extract proteins from cells, RIPA buffer was utilized, supplemented with 1% Roche protease and 1% Sigma phosphatase inhibitor cocktail. The BCA assay was used to calculate the samples' protein concentration. The protein samples were separated via sodium dodecyl sulfate–polyacrylamide gel electrophoresis (SDS-PAGE) and subsequently transferred onto polyvinylidene fluoride (PVDF) membranes (Millipore). After sealing with 5% skim milk, we will crop the membrane according to the molecular weight of the detected protein (such as DBF4 with a molecular weight of 77 kDa, we will crop below 70 kDa and above 100 kDa according to standard protein marker), then detect the membrane with the first antibody, and incubate with the second antibody connected to HRP. Western blot images were captured using the Bio-Rad GelDoc equipment, and signal detection was performed using enhanced ECL. For semi-quantitative analysis, ImageJ software (ImageJ 1.53 k) was employed. We normalized GAPDH as the internal reference protein to calculate the protein levels.

### Statistical analysis

Differential analysis of the gene counts across various samples was performed using the DESeq2 software (DESeq2 1.30.1; URL: https://bioconductor.org/packages/DESeq2/)^22^, employing a filtering criterion of Fold Change ≥ 2 and FDR < 0.01. The fold change represents the ratio of expression levels between two samples (groups). The False Discovery Rate (FDR) is a correction applied to the p-values of significant differences, indicating the significance of the observed differences. To facilitate comparison, the fold change values were logarithmically transformed and expressed as log2FC, wherein larger absolute log2FC values correspond to more pronounced differential changes between the two groups. A volcano plot depicting the differential gene expression analysis results was generated. Gene Set Enrichment Analysis (GSEA) was conducted using the gene sets from the KEGG pathway as the set of genes of interest^23–25^. The enrichment of the gene set of interest was analyzed by scoring it against the background gene set using the log2FC values of each differential group. Data analyses used GraphPad Prism version 9. The data were presented as mean ± standard error, with individual values represented on the scatter plot of the bar graph, and analyzed using a two-sided t-test. Differences were considered significant when *p* < 0.05 and are indicated as ns, not significant, *p < 0.05, **p < 0.01, ***p < 0.001, ****p < 0.0001.

Table 1 provided the full cancer type name corresponding to each abbreviation listed in the legend and the text.

**Table 1:** Full names and abbreviations of the 33 cancers in the TCGA data.

Abbreviation	Full name
ACC	Adrenocortical carcinoma
BLCA	Bladder urothelial carcinoma
BRCA	Breast invasive carcinoma
CESC	Cervical squamous cell carcinoma and endocervical adenocarcinoma
CHOL	Cholangiocarcinoma
COAD	Colon adenocarcinoma
DLBC	Lymphoid neoplasm diffuse large B-cell lymphoma
ESCA	Esophageal carcinoma
GBM	Glioblastoma multiforme
HNSC	Head and neck squamous cell carcinoma
KICH	Kidney chromophobe
KIRC	Kidney renal clear cell carcinoma
KIRP	Kidney renal papillary cell carcinoma
LAML	Acute myeloid leukemia
LGG	Brain lower grade glioma
LIHC	Liver hepatocellular carcinoma
LUAD	Lung adenocarcinoma
LUSC	Lung squamous cell carcinoma
MESO	Mesothelioma
OV	Ovarian serous cystadenocarcinoma
PAAD	Pancreatic adenocarcinoma
PCPG	Pheochromocytoma and paraganglioma
PRAD	Prostate adenocarcinoma
READ	Rectum adenocarcinoma
SARC	Sarcoma
SKCM	Skin cutaneous melanoma
STAD	Stomach adenocarcinoma
TGCT	Testicular germ cell tumors
THCA	Thyroid carcinoma
THYM	Thymoma
UCEC	Uterine corpus endometrial carcinoma
UCS	Uterine carcinosarcoma
UVM	Uveal melanoma

### Ethics approval and informed consent

The Ethics Committee of Renmin Hospital of Wuhan University approved the study. Patients involved obtained informed consent in the study. We confirmed that all methods were carried out by relevant guidelines and regulations.

## Results

### mRNA expression levels and prognostic potential of DBF4 in various cancers

Initially, we used the TCGA dataset to detect differential expression of DBF4 between various tumor types and normal tissues. The results showed that compared with adjacent tissues, the expression of DBF4 was significantly increased in several tumors, including LIHC, BLCA, BRCA, etc. (Fig. S1A, S1B). In addition, we investigated the expression changes of DBF4 using the Oncomine database. The results showed that the expression of DBF4 was significantly up-regulated in liver cancer, brain and central nervous system cancer, head and neck cancer and other cancers (Fig. S1C). In summary, these findings indicate that the expression of DBF4 is frequently upregulated in various tumors, indicating its potential involvement in tumor occurrence and disease progression.

To evaluate the relationship between DBF4 expression and the prognosis of tumor patients, we conducted Cox analysis on OS, DSS, PFI, and DFI using the TCGA database. The results showed the following patterns: DBF4 was identified as a harmful prognostic factor for LIHC, ACC, KICH, KIRC, KIRP, LGG, etc. (Figs. S2A, S2B, S3A, S3B). According to Kaplan Meier analysis, setting the median expression of DBF4 as a threshold, our study found that high levels of DBF4 expression are associated with adverse survival outcomes in several cancers, including LIHC, ACC, KIRC, KIRP, LGG, etc. (Figs. S2A, S2B, S3A, S3B).

In addition, we used a Kaplan Meier plotter to investigate the relationship between DBF4 expression and RFS in different tumor patients. Our analysis shows that in patients with LIHC, PAAD, PCPG, and severe acute respiratory syndrome, elevated DBF4 expression levels are significantly correlated with decreased RFS (Fig. S3C).

### Validating the expression and prognostic value of DBF4 in HCC

**Figure 1:**
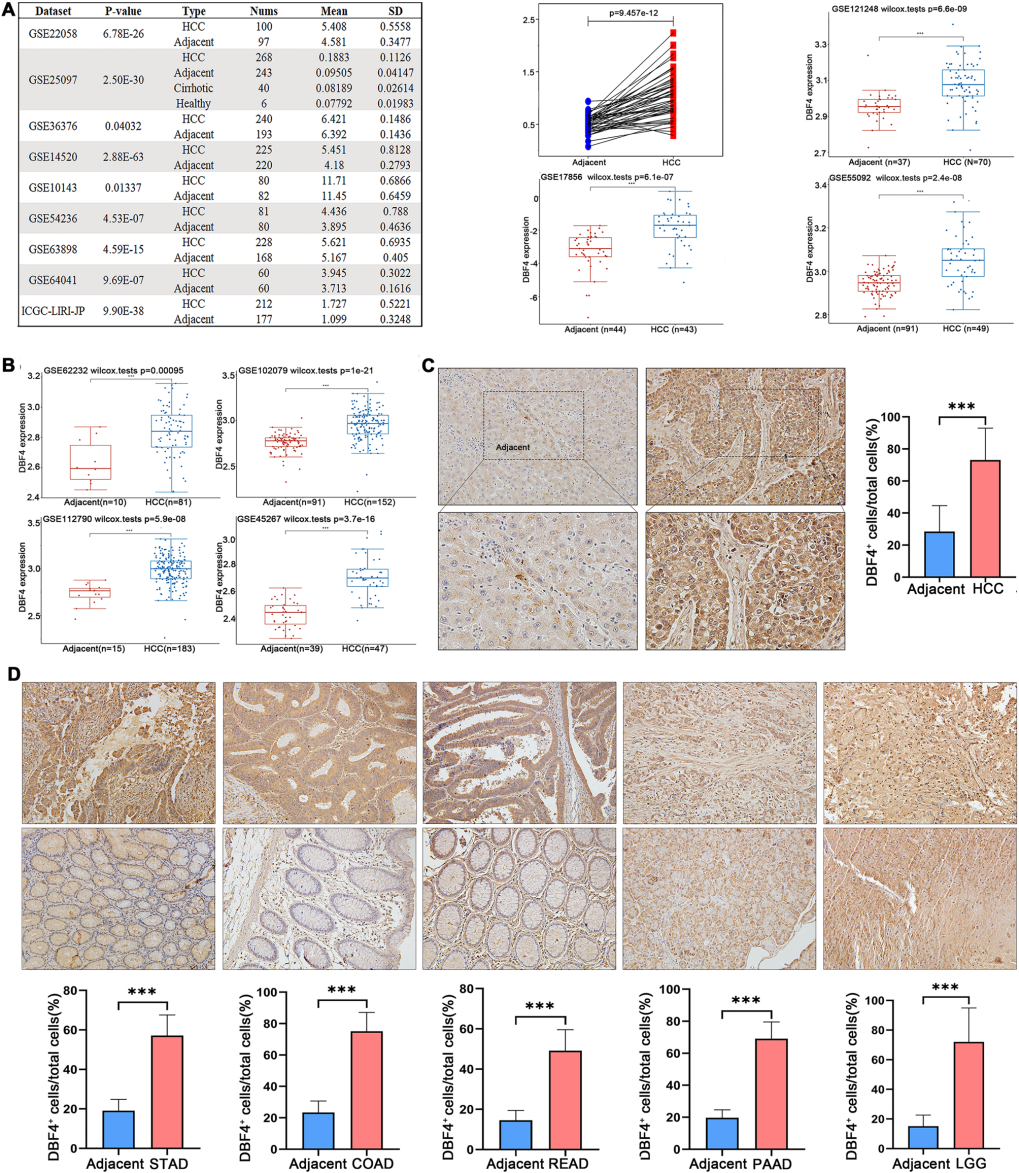
Validating the expression of DBF4 in HCC. Validation of DBF4 expression by HCCDB database, TCGA, GSE121248, GSE102079, GSE62232, GSE17856, GSE55092, GSE45267, and GSE112790 (**A,B**). (**C**) Representative Immunohistochemistry figures and variance analysis in HCC (200x). (**D**) Representative Immunohistochemistry figures and variance analysis in STAD, COAD, READ, PAAD, and LGG (× 200). (*p < 0.05, **p < 0.01, ***p < 0.001).

The close association between DBF4 and the prognosis of HCC prompted us to conduct a comprehensive investigation into their relationship. To achieve this, we utilized multiple datasets including the TCGA dataset (HCC paired data), the HCCDB database (GSE10143, GSE14520, etc.), GSE102079, GSE112790, GSE121248, GSE17856, GSE45267 and others. These datasets were employed to validate the disparity in DBF4 expression. Our findings consistently demonstrated a significant upregulation of DBF4 expression in HCC compared to aparacancerous tissue (Fig. 1A,B). Then, we performed IHC to assess the protein level of DBF4. The IHC analysis confirmed a remarkable increase in DBF4 expression in HCC (Fig. 1C). The DBF4 expression levels correlate closely with tumor size (*P* = 0.002), vascular invasion (*P* = 0.048) and TMN stage (*P* = 0.034) (Table S1). Similar trends were also observed in other cancer types such as STAD, READ, COAD, PAAD, and LGG (Fig. 1D).

**Figure 2:**
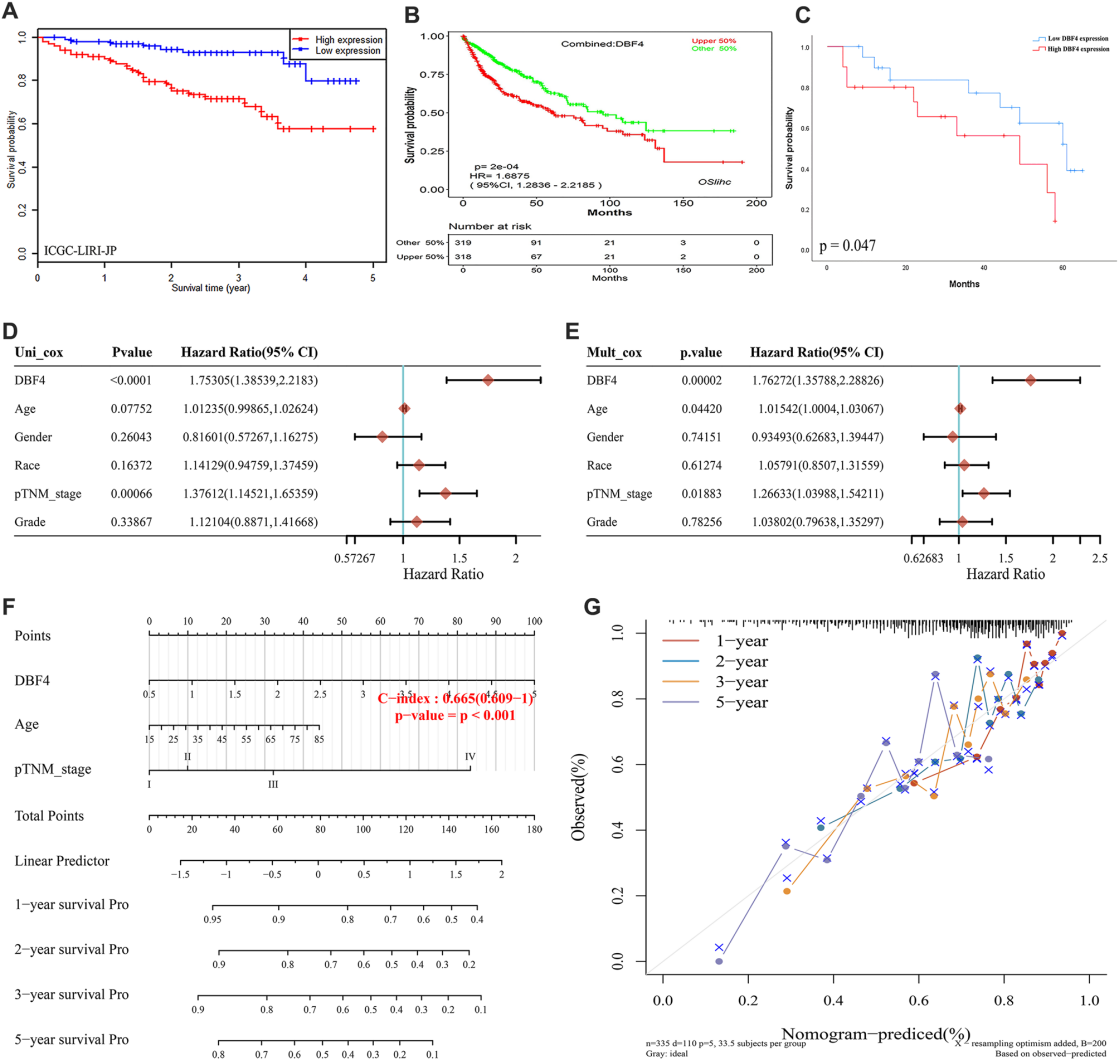
Validating the prognostic value of DBF4 in HCC. (**A**) LIRI-JP dataset was used to verify the poor prognosis of DBF4 in HCC patients. (**B**) The combined dataset (combined TCGA, GSE20140, GSE27150, GSE76427 datasets) was used to verify the poor prognosis of DBF4 in HCC patients. (**C**) Prognostic analysis of survival data of the 40 patients with liver cancer we included according to median DBF4 expression. (**D,E**) Hazard ratio and *P* value of constituents involved in univariate and multivariate Cox regression and some parameters of the DBF4 genes [Male vs. Female (reference values); Race: Black/White vs. Asian (reference values); pTNM stage: III/IV vs. I/II (reference values); Grade: G3/G4 vs. G1/G2 (reference values)]. (**F**) Nomogram to predict the 1-year, 2-year, 3-year, and 5-year overall survival of HCC cancer patients. (**G**) Calibration curve for the overall survival nomogram model. A dashed diagonal line represents the ideal nomogram, and the red line, blue line, orange line, and purple line represent the 1-year, 2-year, 3-year, and 5-year observed nomograms.

We conducted analyses using the ICGC-LIRI-JP dataset, as well as the combined dataset (combined TCGA, GSE20140, GSE27150, GSE76427 datasets). The survival data of more than 40 liver cancer patients were also included in this investigation. Both of which showed a strong correlation between high DBF4 expression and poor prognosis in HCC patients (Fig. 2A–C). With the aim of establishing the reliability of DBF4 as a prognostic predictor for HCC patients, we further examined the TCGA data. As shown in Fig. 2D and E, In Cox regression analysis, both in the univariate and multivariate models, revealed a robust association between DBF4 expression and OS in HCC. The expression level of DBF4 is a reliable predictor of poor prognosis in HCC. Taking into account DBF4 expression and clinicopathological features, we employed nomogram predictive models to accurately estimate the probability of 1-, 2-, 3-, and 5-year OS in patients with HCC. The predictive models demonstrated a satisfactory concordance index (C-index: 0.665, 95% confidence interval: 0.609–1) with a significant statistical significance (*p* < 0.001), that provided a clinically applicable method that could predict the survival probability of patients with HCC (Fig. 2F). The calibration plots for the 1-, 2-, and 3-year OS rates exhibited close agreement with the ideal model (Fig. 2G).

### Knockdown of DBF4 inhibits proliferation, migration, and invasion of HCC cells

**Figure 3:**
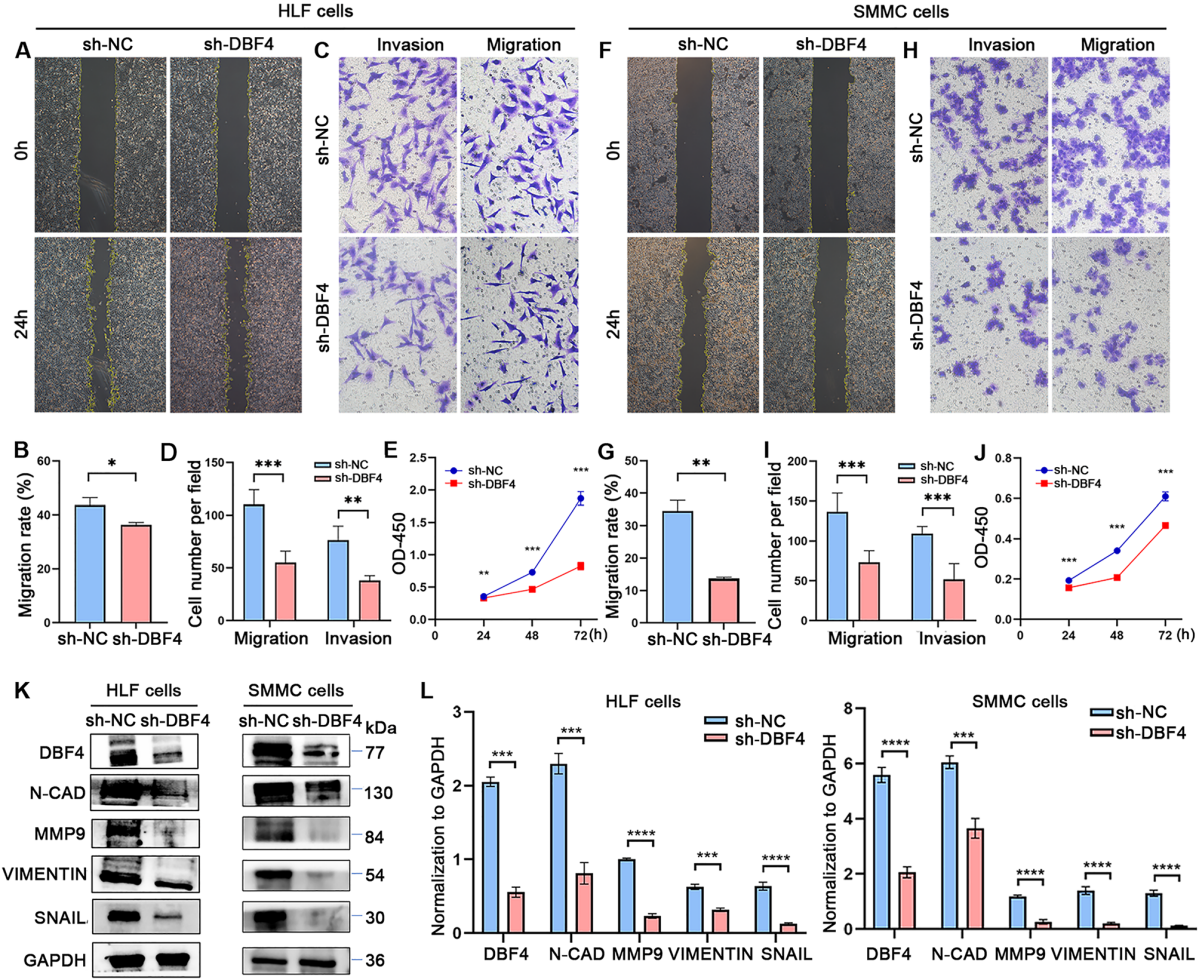
The cell proliferation, migration, and invasion were suppressed in HLF and SMMC-7721 cells as a result of DBF4 knockdown. Knockout of DBF4 reduced the migration ability of HLF (**A,B**) and SMMC-7721 (**F,G**) cells. Migration and invasion assays were performed to estimate the migratory and invasive capabilities of HLF (**C,D**) and SMMC-7721 (**H,I**) cells transfected with lentivirus-mediated transfection. The CCK-8 analysis revealed that the proliferative ability was inhibited in HLF (**E**) and SMMC-7721 (**J**). The WB results showed a decrease in the expression of EMT-related proteins (**K,L**). (*p < 0.05, **p < 0.01, ***p < 0.001, ****p < 0.0001).

We explored the effects of DBF4 on HCC cells in vitro. Initially, we constructed DBF4 knockout HLF and SMMC-7721 cells using lentivirus-mediated transfection method. The successful knockdown in post-transfection HLF and SMMC cells was subsequently confirmed by WB analysis (Fig. S4A, S4B). The results of wound-healing assay exposed that the cell scratch areas healed was remarkably decreased after knocking down the expression of DBF4 in HLF and SMMC-7721 cells (Fig. 3A,B,F,G); The transwell migration and invasion assay results demonstrate that the number of cells crossing the upper and lower chamber membranes in HLF and SMMC-7721 significantly decreased after knocking down the expression of DBF4 (Fig. 3C,D,H,I); CCK-8 results showed that the OD values of cells at 24, 48 and 72 h were significantly lower after knockdown of DBF4 in the HLF and SMMC-7721 (Fig. 3E,J). Moreover, we conducted the impact of DBF4 on epithelial-mesenchymal transition (EMT). After knocking down DBF4, the expression levels of N-CAD, MMP9, VIMENTIN and SNAIL, were significantly reduced (Fig. 3K,L).

### Overexpression of DBF4 promotes proliferation, migration, and invasion of HCC cells

**Figure 4:**
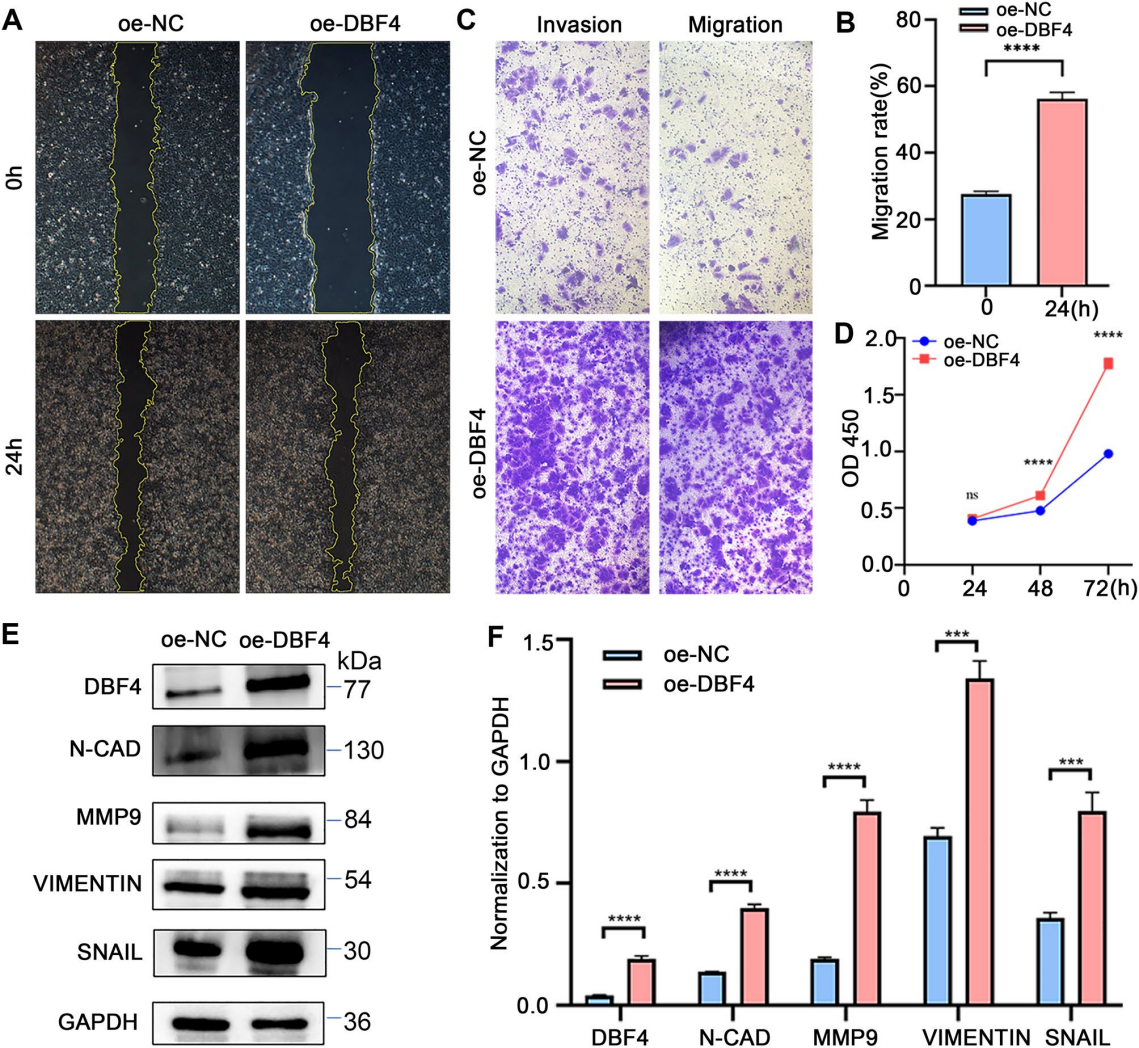
Overexpression of DBF4 enhances proliferation, migration, and invasion of Huh7 cells. The overexpression of DBF4 enhances the migratory ability of Huh7 cells (**A,B**). Migration and invasion assays were performed to assess the migratory and invasive capabilities of Huh7 cells overexpressing DBF4 (**C**). CCK-8 analysis revealed enhanced proliferation ability in Huh7 cells (**D**). Western blot results demonstrated an upregulation in the expression of EMT-related proteins (**E,F**). (^ns^*p* > 0.05, ***p < 0.001, ****p < 0.0001).

Subsequently, DBF4 overexpression in Huh7 cells was achieved through lentivirus-mediated transfection (Fig. S4A, S4B). The results of the wound-healing assay exhibited a significant increase in the area of cell migration following DBF4 overexpression in Huh7 cells (Fig. 4A,B). The outcomes of the transwell migration and invasion experiments further indicated a substantial augmentation in the quantity of Huh7 cells transversing the upper and lower chambers upon DBF4 overexpression (Fig. 4C). Additionally, the CCK-8 assays revealed a marked elevation in the OD values at 24, 48, and 72 h post-DBF4 overexpression in Huh7 cells (Fig. 4D). The upregulation of N-CAD, MMP9, VIMENTIN, and SNAIL expression levels was also significantly observed subsequent to DBF4 overexpression (Fig. 4E,F).

### KEGG pathway analysis of DBF4 in HCC

**Figure 5:**
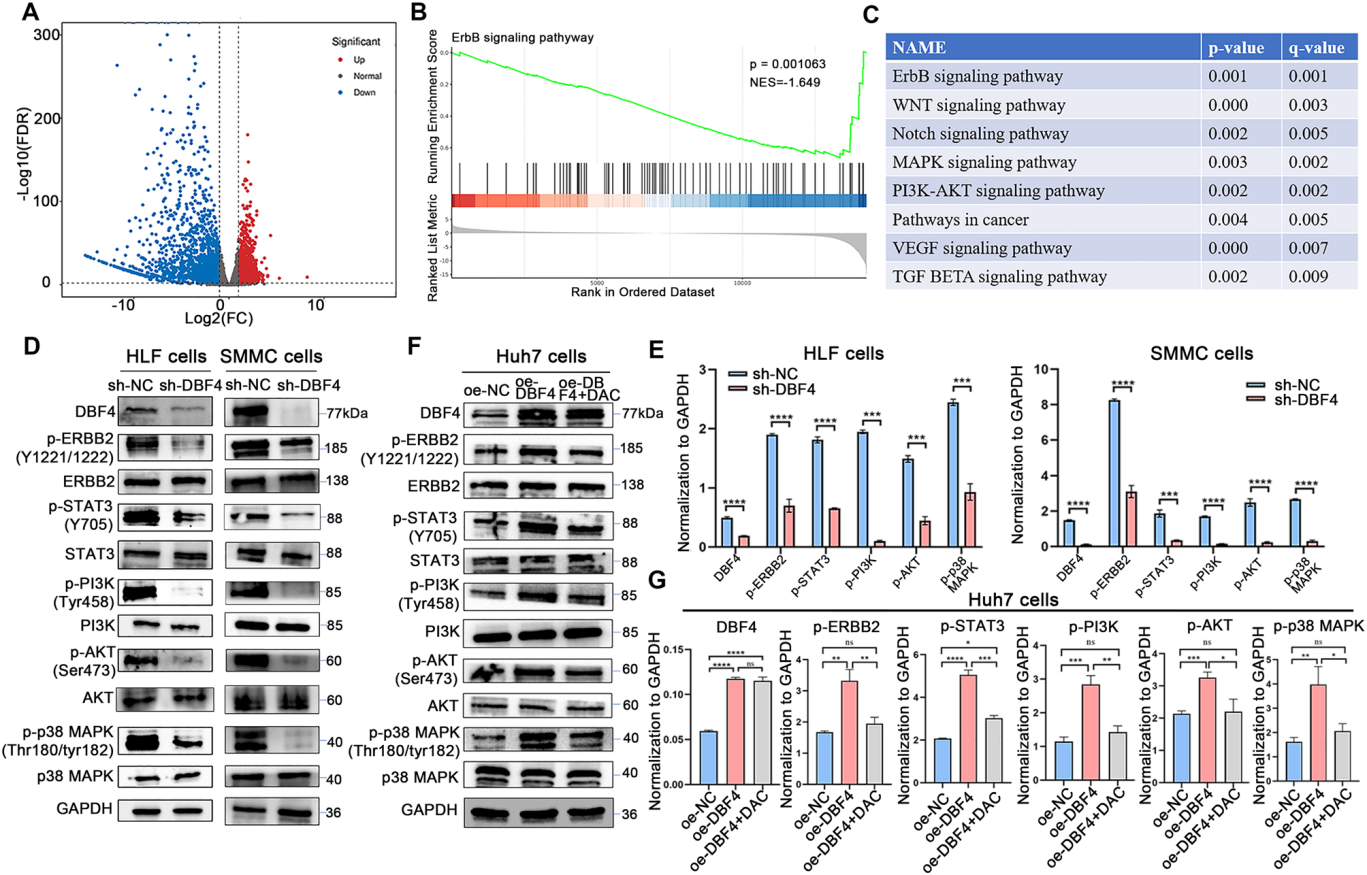
Validating the potential signaling pathways of DBF4. A volcano plot was constructed to visualize the differential gene expression after knockdown of DBF4 in cells (**A**). KEGG pathway analysis after sequencing (**B**–**C**). WB results demonstrated changes in ERBB2, p-PI3K, p-Akt, p-STAT3 and p-p38 MAPK signaling pathways following knockout of DBF4 (**D,E**) or overexpression of DBF4 (**F,G**). (^ns^*p* > 0.05, *p < 0.05, **p < 0.01, ***p < 0.001, ****p < 0.0001).

To elucidate the potential mechanisms by which DBF4 exerts its effects in tumor cells, we compared the transcriptomes of HLF cells transfected with sh-NC and sh-DBF4. The results revealed that, in comparison to the control group, HLF cells with DBF4 knockdown displayed differential expression in 3250 genes. Among these genes, 1782 were downregulated, and 1468 were upregulated, with the downregulated genes exhibiting more pronounced differential expression (Fig. 5A). Then we performed Gene Set Enrichment Analysis to identify the enriched KEGG signaling pathway. The results explored that DBF4 was mostly enriched in ERBB signaling pathway (*p* = 0.001, FDR = 0.001), WNT signaling pathway (*p* < 0.001, FDR = 0.003), Notch signaling pathway (*p* = 0.002, FDR = 0.005), MAPK signaling pathway (*p* = 0.003, FDR = 0.002), PI3K-AKT signaling pathway (*p* = 0.002, FDR = 0.002), pathways in cancer (*p* = 0.004, FDR = 0.005), VEGF signaling pathway (*p* < 0.001, FDR = 0.007), TGF-BETA signaling pathway (*p* = 0.002, FDR = 0.009) and suggesting that DBF4 is indeed involved in tumor development (Fig. 5B,C). The ERBB family of proteins comprises ERBB1 (also known as EGFR), ERBB2, ERBB3, and ERBB4. The downstream signaling pathways of the ERBB family are interconnected and overlapping, including the PI3K/AKT, MAPK, JAK/STAT3, and other signaling pathways^26–33^. Based on this, we further validated the impact of DBF4 knockdown on cell signaling pathways using WB experiments. The results demonstrated that ERBB2 was significantly inhibited upon DBF4 knockdown in HLF and SMMC-7721 cells, and the expression of p-PI3K, p-Akt, p-p38 MAPK and p-STAT3 were also significantly decreased (Fig. 5D,E). In addition, we also analyzed the changes in the EGFR signaling pathway after intervention with DBF4 through WB experiments. The results showed that overexpression of DBF4 increased the activation level of the EGFR signaling pathway. On the contrary, knocking down DBF4, the activation of the EGFR signaling pathway was inhibited (Fig. S5A, S5B).

### Validating the impact of DBF4 on the ERBB pathway

**Figure 6:**
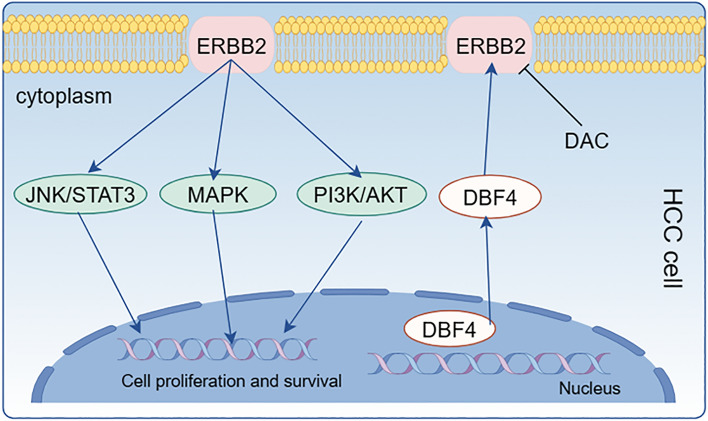
The model of DBF4 in the carcinogenesis of HCC.

**Figure 7:**
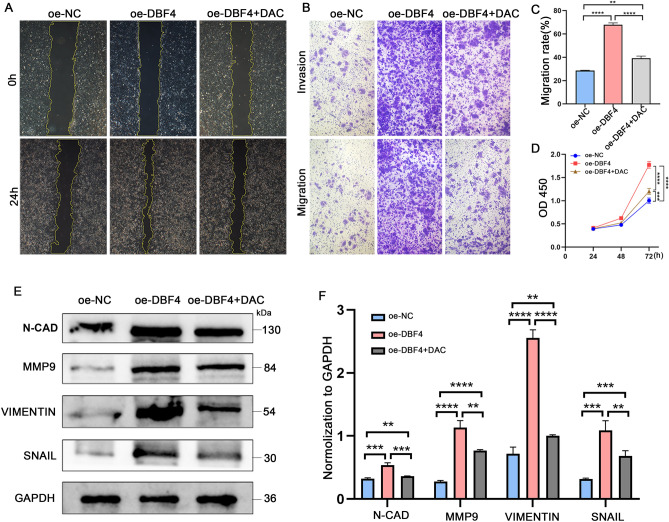
ERBB inhibitors rescue proliferation, migration, and invasion promoted by overexpression of DBF4 of Huh7 cells. The migratory and invasive abilities of HLF cells overexpressing DBF4 were attenuated upon treatment with ERBB inhibitor (**A**–**C**). CCK-8 analysis showed that inhibition of ERBB drastically suppressed the proliferation ability of DBF4-overexpressing HLF cell (**D**). Western blot results showed that the expression of EMT related proteins was downregulated after treatment with ERBB inhibitors (**E,F**). (**p < 0.01, ***p < 0.001, ****p < 0.0001).

In order to further ascertain the effect of DBF4 on HCC cells through the ERBB pathway, we subjected a part of Huh7 cells overexpressing DBF4 to treatment with the ERBB inhibitor dacomitinib for 24 h. Following this, protein was extracted for WB experiments to observe the patterns of protein expression. The results reveal that, following the inhibition of ERBB2, there is a notable decline in the expression levels of the p-PI3K, p-Akt, p-p38 MAPK and p-STAT3 (Fig. 5F,G). The findings indicate that DBF4 has the ability to activate downstream signaling pathways (PI3K/AKT, MAPK, JAK/STAT3) by promoting the ERBB signaling pathway (Fig. 6). Subsequently, to investigate the effects of suppressing ERBB2 on the proliferation, migration, and invasion capabilities of liver cancer cells, we proceeded with additional cellular functional experiments. The results of the wound-healing assay revealed a significant decrease in the healed scratch area of Huh7 cells overexpressing DBF4 after treatment with dacomitinib (Fig. 7A,C). The transwell migration and invasion assay demonstrated a notable reduction in the number of Huh7 cells overexpressing DBF4 that crossed the upper and lower chambers following dacomitinib treatment (Fig. 7B). Moreover, the CCK-8 assay indicated a significant decrease in the OD values at 24, 48, and 72 h in Huh7 cells overexpressing DBF4 after treatment with dacomitinib, as compared to the untreated group (Fig. 7D). And after using ERBB inhibitor dacomitinib, there was a significant decrease in N-CAD, MMP9, VIMENTIN, and SNAIL expression levels (Fig. 7E,F).

## Discussion

The precise role of DBF4 in tumor development is currently under investigation, with most studies focusing on the function of the DBF4 and CDC7 kinase complex (DDK kinase)^2,34^, with few reports on DBF4 alone. To gain deeper insights into the functional significance of DBF4 in different malignancies, we conducted a comprehensive and systematic analysis of DBF4 across various tumors. Initially, we examined multiple datasets and observed that DBF4 expression was consistently upregulated in the majority of tumors. To validate these findings, we further analyzed human tissue samples from HCC, STAD, READ, COAD, PAAD, and LGG. Strikingly, in some tumors such as HCC, the higher the histological grade, the later the clinical stage, and the higher the expression level of DBF4. These observations suggest a potential role for DBF4 in promoting tumor progression and metastasis.

In our study, we observed a significant association between increased DBF4 expression and poor OS, DSS, DFI, PFI, and RFS, particularly in HCC. To further validate the prognostic value of DBF4 expression in HCC, we analyzed multiple datasets and confirmed that DBF4 expression could serve as an independent prognostic factor for HCC patients. Overall, our analysis across pan-cancer revealed that DBF4 is overexpressed in the majority of cancer types, and is associated with unfavorable prognosis, especially notable in HCC. Our team possesses a substantial collection of HCC specimens coupled with extensive research experience in the field^35,36^. Using these HCC samples, we proceeded to validate the correlation between high DBF4 expression and poor prognosis in HCC. Therefore, we undertook a comprehensive study on the impact of DBF4 on the progression of HCC and the potential underlying mechanisms.

Subsequent to knocking out DBF4, there was a noticeable decrease in the proliferation, migration, and invasive activity of liver cancer cells. Conversely, overexpression of DBF4 resulted in a significant enhancement in the proliferation, migration, and invasive capabilities of liver cancer cells. In order to gain a deeper understanding of the potential mechanisms by which DBF4 promotes cancer progression, we conducted cellular transcriptomic and KEGG enrichment analyses. Our findings suggest that DBF4 may regulate tumor progression by influencing the ERBB signaling pathway and its downstream pathways including JNK/STAT3, MAPK, and PI3K/AKT signaling pathways. Cancer is a complex disease characterized by coordinated changes in various signaling pathways. Previous studies have shown that genetic alterations in the ERBB gene family can aberrantly activate the ERBB1 and ERBB2 signals, leading to tumor initiation, growth, and progression^33^. Abnormal activation of the JNK/STAT3 signaling pathway has been observed in multiple types of tumors, promoting tumor proliferation and angiogenesis^32^. The PI3K/AKT pathway is a complex signaling pathway composed of multiple upstream regulatory factors and downstream effectors, and its overactivation can facilitate tumor development^37^. The MAPK signaling pathway has been extensively studied in cancer biology, with over 40% of human cancer cases attributed to its hyperactivation^26^. It appears plausible that DBF4 drives tumor progression by modulating these key pathways. Thus, we performed WB experiments and observed a significant decrease in the expression levels of ERBB2 and its downstream signaling pathways following DBF4 knockdown, while overexpression of DBF4 yielded opposite results.

To confirm the impact of DBF4 on liver cancer cells through the ERBB signaling pathway, we introduced the ERBB inhibitor dacomitinib after DBF4 overexpression. Interestingly, after treatment with ERBB inhibitors, the expected increase in proliferation, migration, and invasion abilities of liver cancer cells were suppressed, accompanied by a notable decrease in the expression levels of ERBB2 and its downstream signaling pathways. These experimental findings provide evidence that DBF4 can promote the progression of liver cancer by activating the ERBB signaling pathway and its downstream cascades.

While our study has investigated the role of DBF4 in various malignant tumors, there are still some limitations. We have confirmed that the expression differences of DBF4 at the tissue level exist in HCC, STAD, COAD, READ, PAAD, and LGG. And the tumor biological effects of DBF4 on HCC, with potential signaling pathways concentrated in ERBB. However, other potential pathways cannot be ruled out, and further research is needed to validate our findings in other tumor types. Secondly, the role of CDC7 in the activation of the ERBB signaling pathway by DBF4 is still unclear and further experiments are needed to verify.

In summary, the activation of the ERBB pathway through DBF4 has been shown to enhance the proliferation, migration, and invasion capabilities of liver cancer cells, thereby promoting tumor progression. Moreover, the overexpression of DBF4 is associated with poor prognosis and various malignant tumors, including HCC, making it a potential independent prognostic indicator in HCC. Therefore, targeting DBF4 interference holds promise as a therapeutic strategy for cancer patients. However, further fundamental research and large-scale clinical trials are required to validate these findings in the future.

### Supplementary Information

Supplementary Information 1.

Supplementary Information 2.

## Data Availability

The dataset analyzed during the current study consists of public datasets such as TCGA, GEO, and information from our hospital patients, which can be downloaded from the methodology section of the manuscript or obtained from the corresponding author upon reasonable request. The metadata generated from sequencing in this study can be accessed in the NCBI database (PRJNA1037690).
