# Supplementary material for: The prognostic significance and potential mechanism of DBF4 zinc finger in hepatocellular carcinoma
Source: Sci Rep. 2024 May 9;14:10662. doi: 10.1038/s41598-024-60342-w (PMC11082141; doi:10.1038/s41598-024-60342-w)

Figure 3K

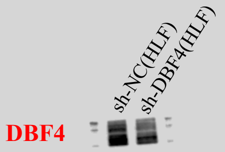

Figure 3K

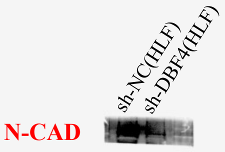

Figure 3K

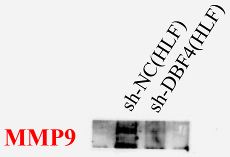

Figure 3K

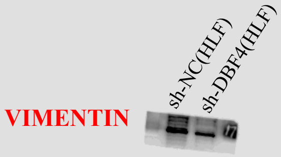

Figure 3K

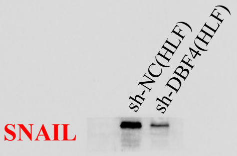

Figure 3K

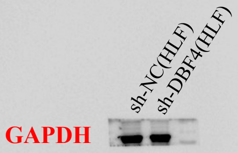

Figure 3K

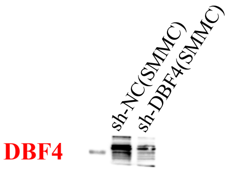

Figure 3K

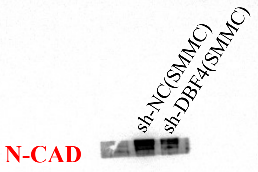

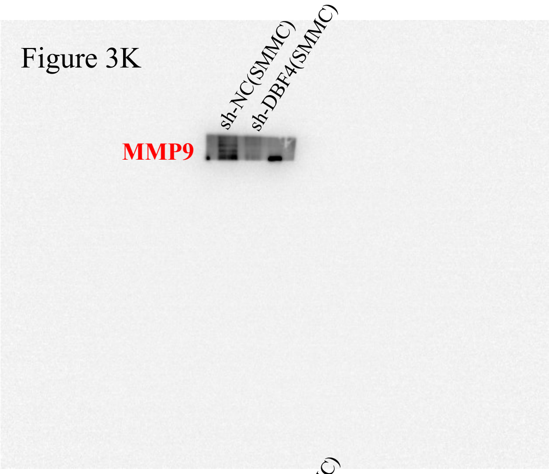
Figure 3K
MMP9
sh-NC(SMMC)
sh-DBF4(SMMC)

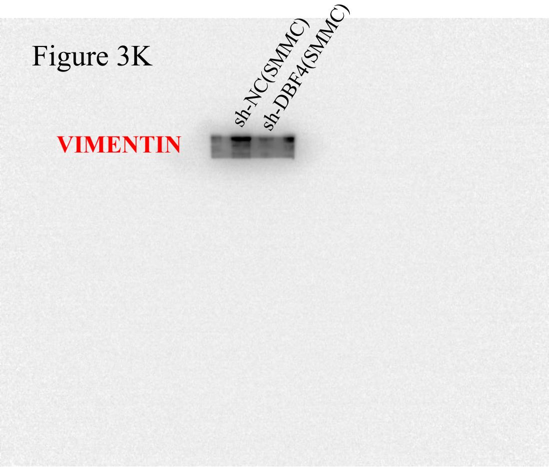
Figure 3K
VIMENTIN
sh-NC(SMMC)
sh-DBF4(SMMC)

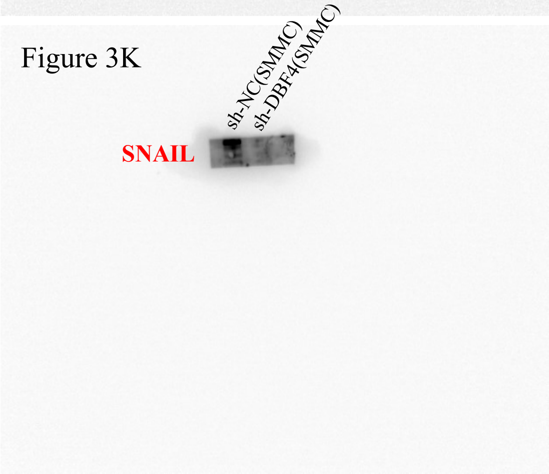
Figure 3K
SNAIL
sh-NC(SMMC)
sh-DBF4(SMMC)

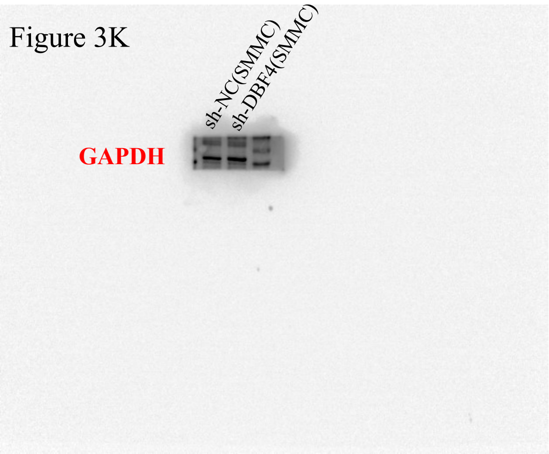
Figure 3K
GAPDH
sh-NC(SMMC)
sh-DBF4(SMMC)

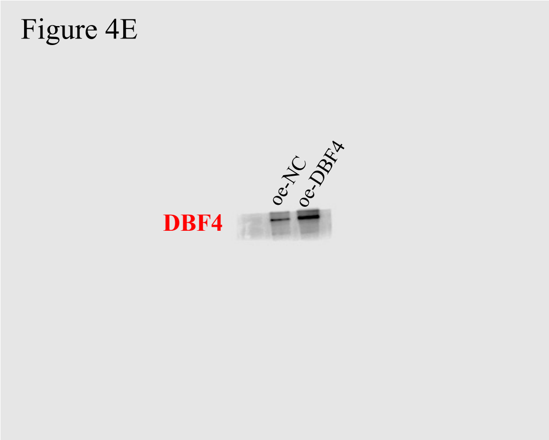
Figure 4E
DBF4
oe-NC
oe-DBF4

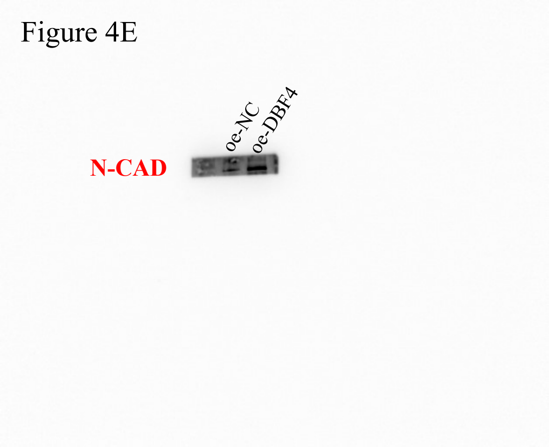
Figure 4E
N-CAD
oe-NC
oe-DBF4

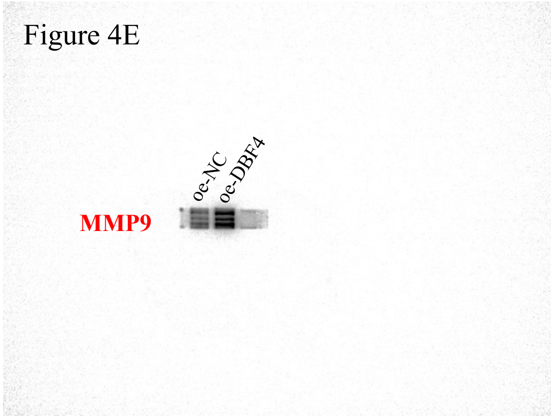
Figure 4E
MMP9
oe-NC
oe-DBF4

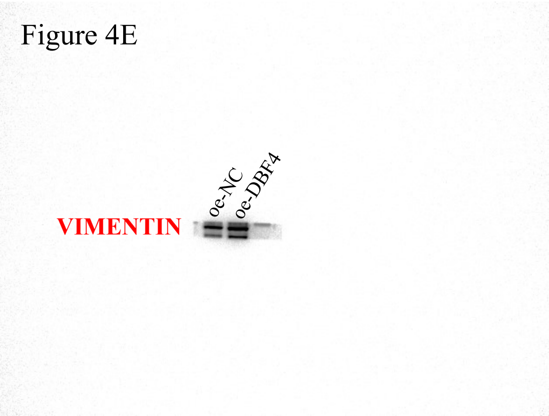
Figure 4E
VIMENTIN
oe-NC
oe-DBF4

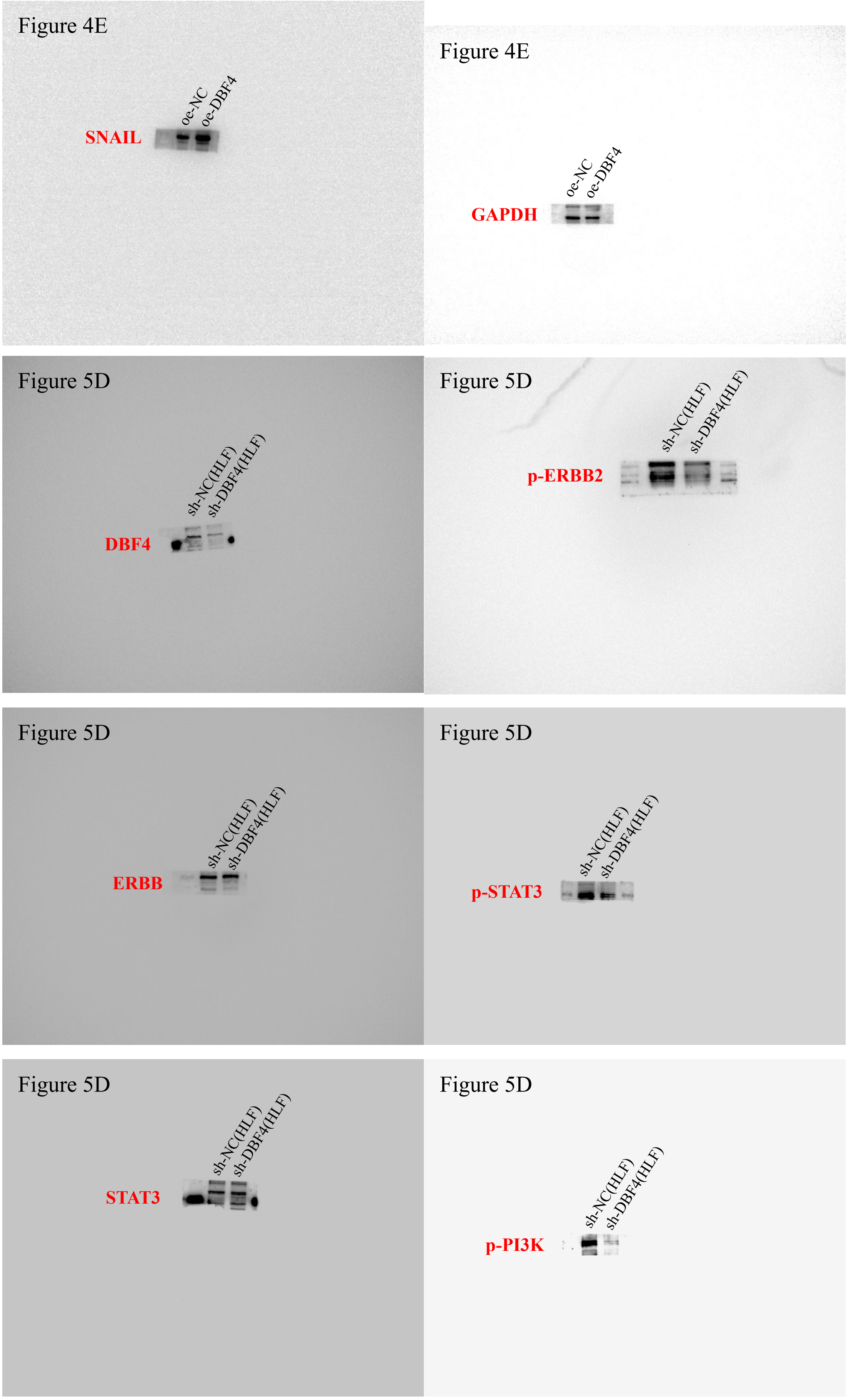
Figure 4E
oe-NC
oe-DBF4
SNAIL
Figure 4E
oe-NC
oe-DBF4
GAPDH
Figure 5D
sh-NC(HLF)
sh-DBF4(HLF)
DBF4
Figure 5D
sh-NC(HLF)
sh-DBF4(HLF)
p-ERBB2
Figure 5D
sh-NC(HLF)
sh-DBF4(HLF)
ERBB
Figure 5D
sh-NC(HLF)
sh-DBF4(HLF)
p-STAT3
Figure 5D
sh-NC(HLF)
sh-DBF4(HLF)
STAT3
Figure 5D
sh-NC(HLF)
sh-DBF4(HLF)
p-PI3K

Figure 5D

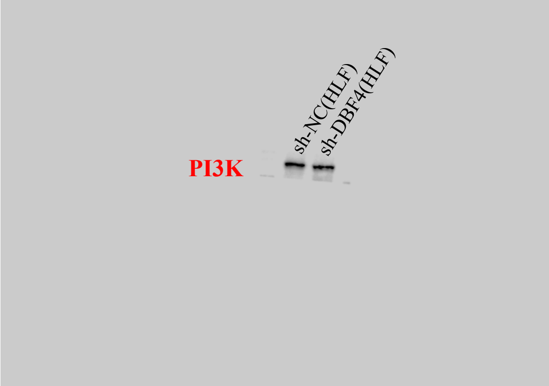

Figure 5D

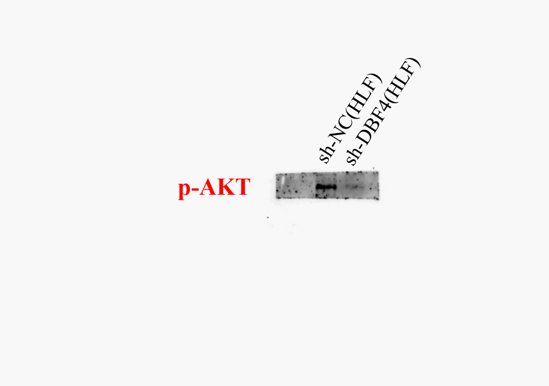

Figure 5D

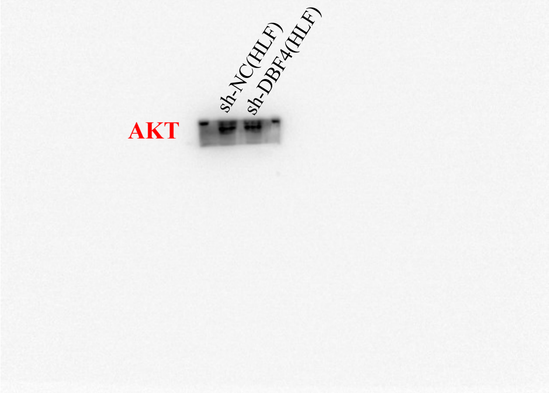

Figure 5D

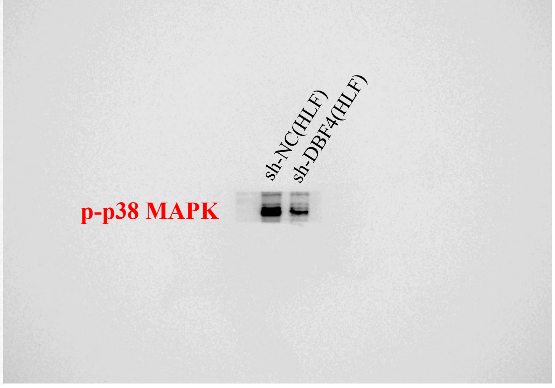

Figure 5D

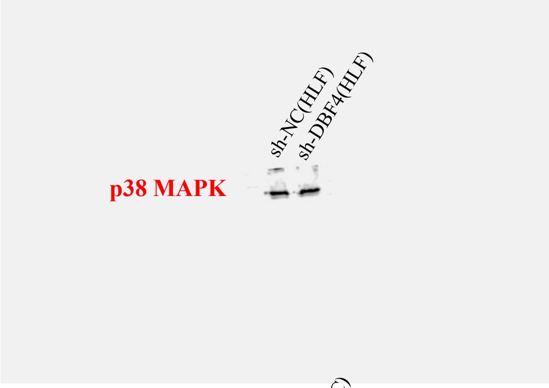

Figure 5D

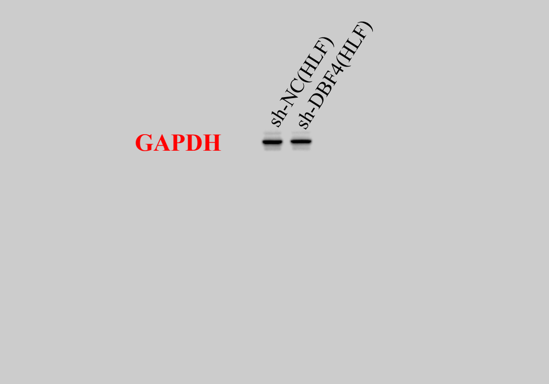

Figure 5D

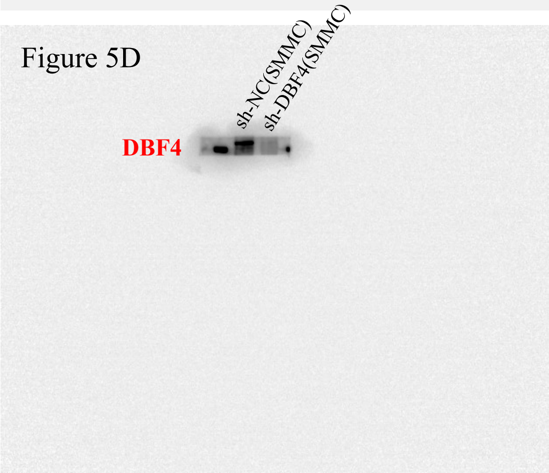

Figure 5D

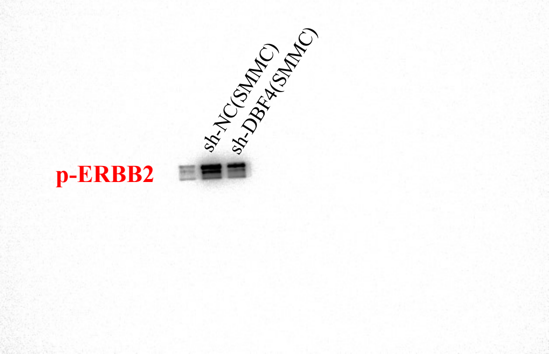

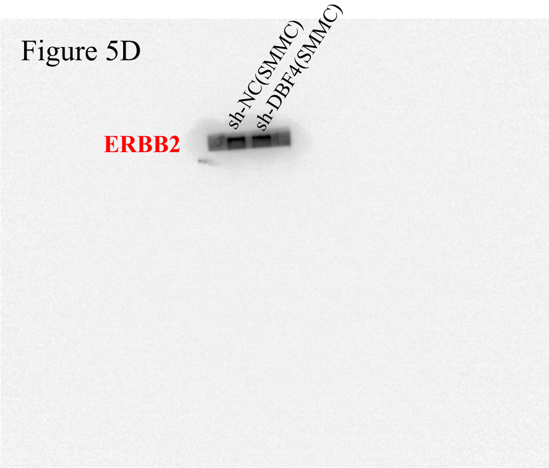
Figure 5D
sh-NC(SMMC)
sh-DBF4(SMMC)
ERBB2

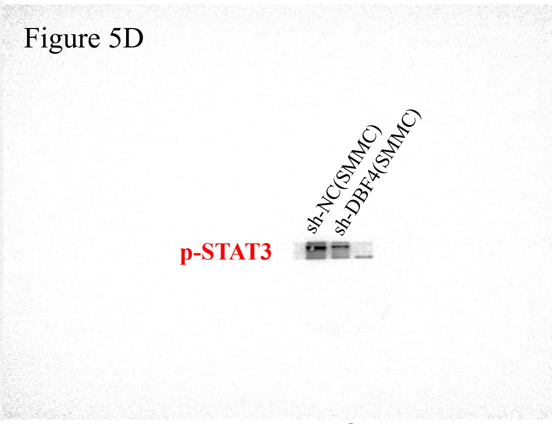
Figure 5D
sh-NC(SMMC)
sh-DBF4(SMMC)
p-STAT3

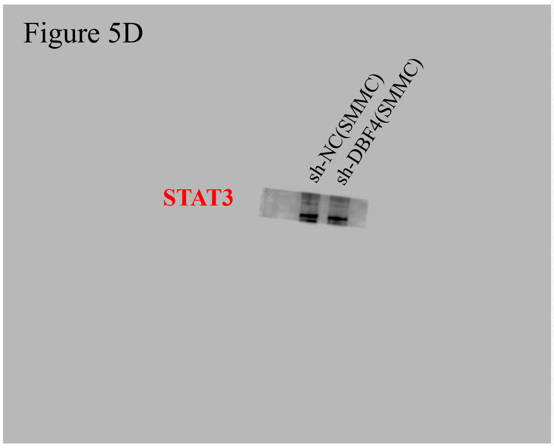
Figure 5D
sh-NC(SMMC)
sh-DBF4(SMMC)
STAT3

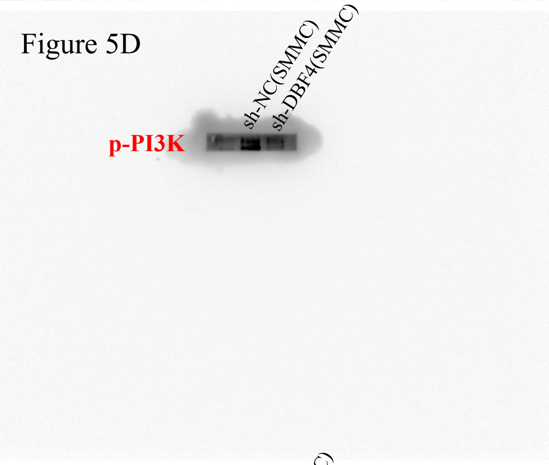
Figure 5D
sh-NC(SMMC)
sh-DBF4(SMMC)
p-PI3K

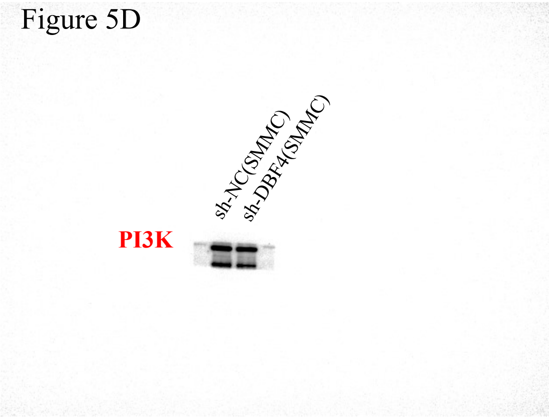
Figure 5D
sh-NC(SMMC)
sh-DBF4(SMMC)
PI3K

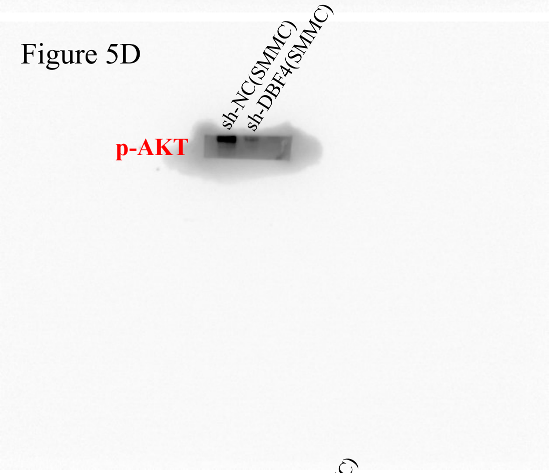
Figure 5D
sh-NC(SMMC)
sh-DBF4(SMMC)
p-AKT

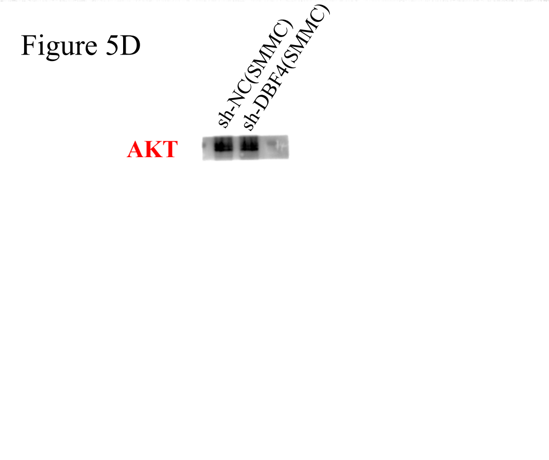
Figure 5D
sh-NC(SMMC)
sh-DBF4(SMMC)
AKT

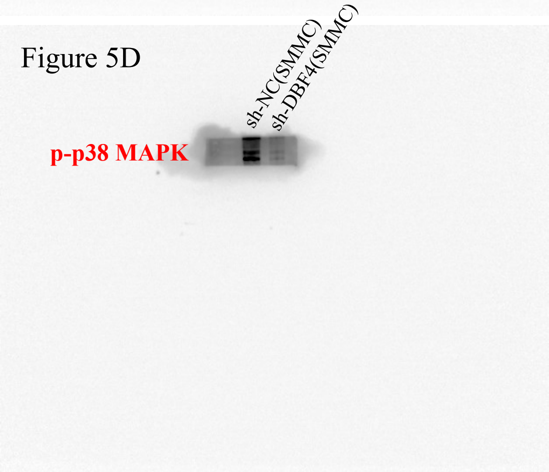
Figure 5D
sh-NC(SMMC)
sh-DBF4(SMMC)
p-p38 MAPK

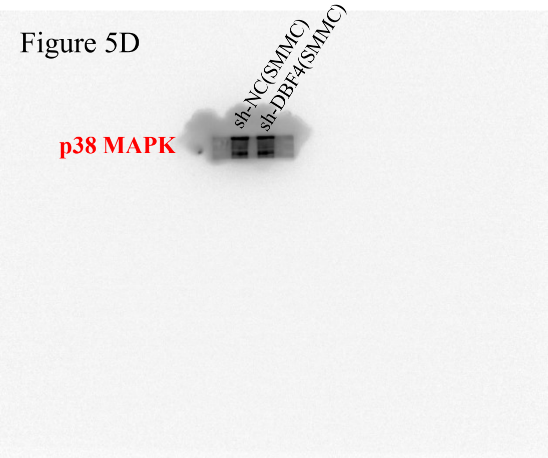
Figure 5D
sh-NC(SMMC)
sh-DBF4(SMMC)
p38 MAPK

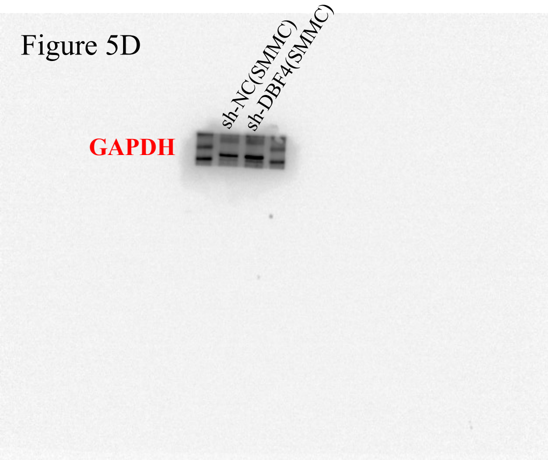
Figure 5D
sh-NC(SMMC)
sh-DBF4(SMMC)
GAPDH

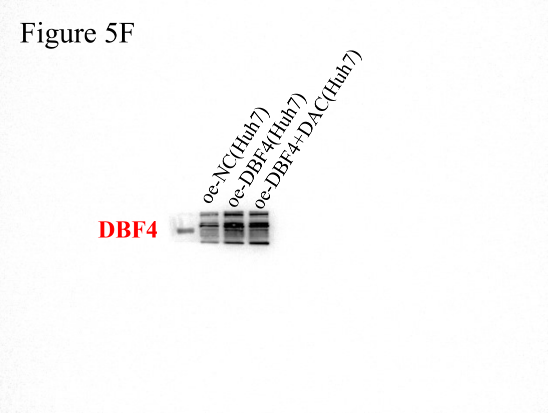
Figure 5F
oe-NC(Huh7)
oe-DBF4(Huh7)
oe-DBF4+DAC(Huh7)
DBF4

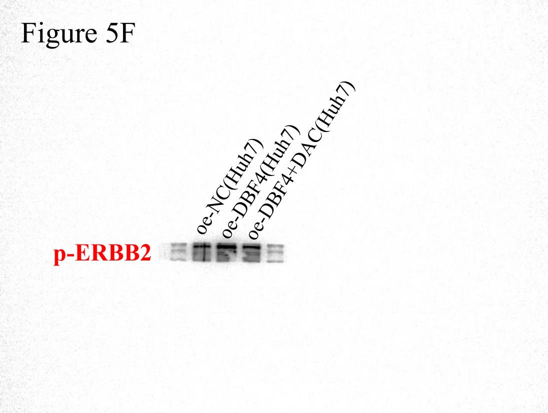
Figure 5F
oe-NC(Huh7)
oe-DBF4(Huh7)
oe-DBF4+DAC(Huh7)
p-ERBB2

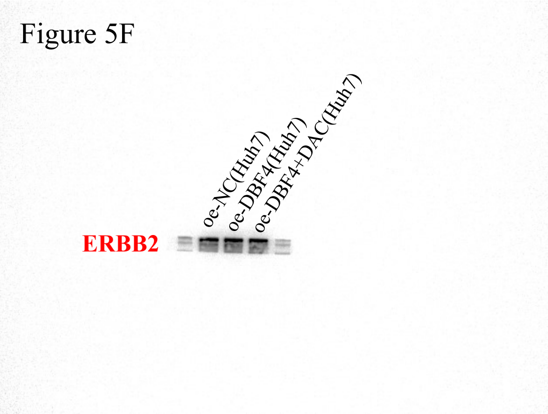
Figure 5F
oe-NC(Huh7)
oe-DBF4(Huh7)
oe-DBF4+DAC(Huh7)
ERBB2

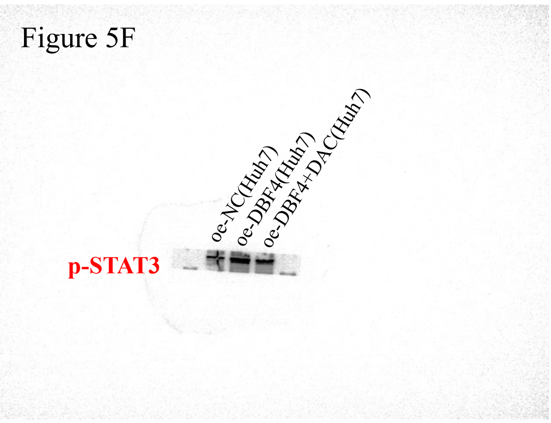
Figure 5F
oe-NC(Huh7)
oe-DBF4(Huh7)
oe-DBF4+DAC(Huh7)
p-STAT3

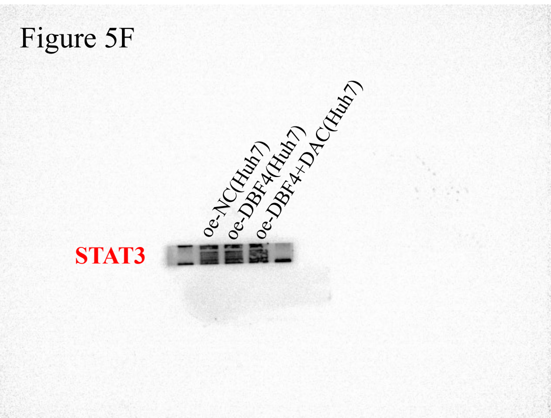
Figure 5F
oe-NC(Huh7)
oe-DBF4(Huh7)
oe-DBF4+DAC(Huh7)
STAT3

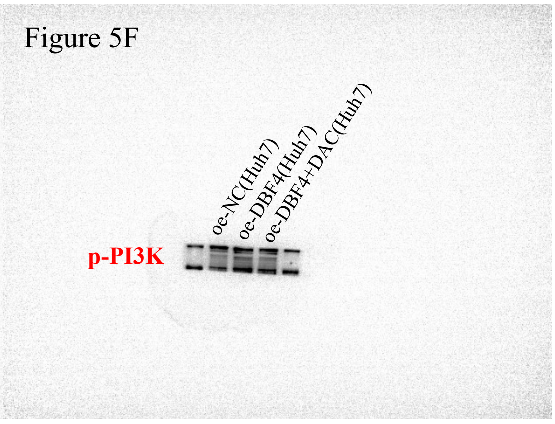
Figure 5F
oe-NC(Huh7)
oe-DBF4(Huh7)
oe-DBF4+DAC(Huh7)
p-PI3K

Figure 5F

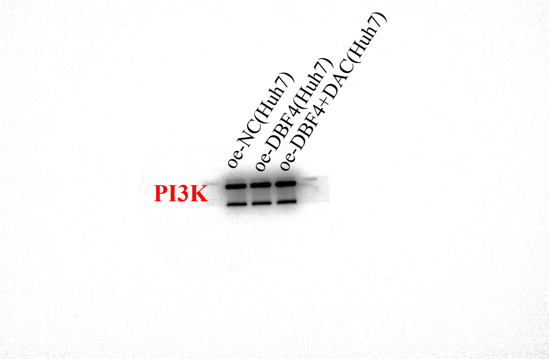

Figure 5F

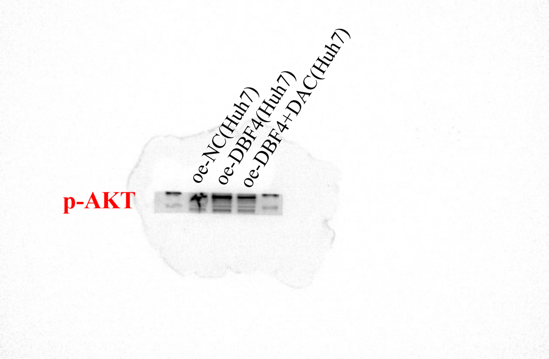

Figure 5F

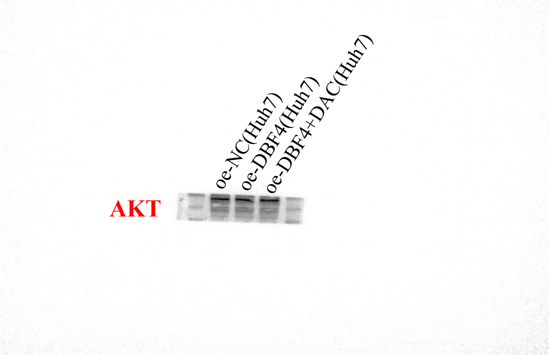

Figure 5F

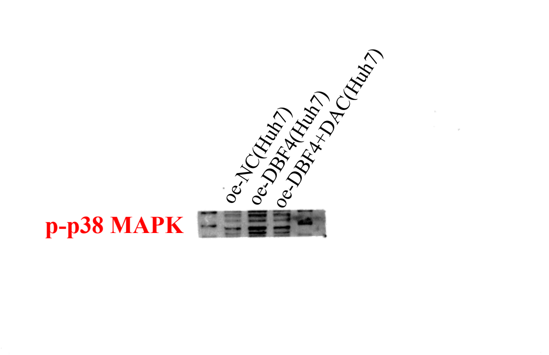

Figure 5F

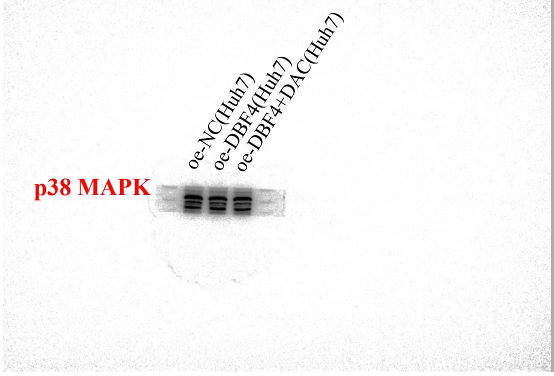

Figure 5F

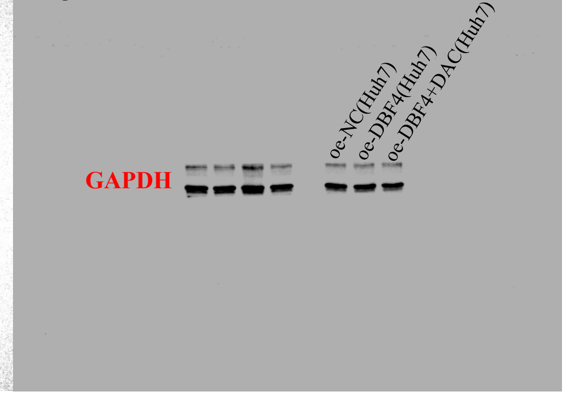

Figure 6E

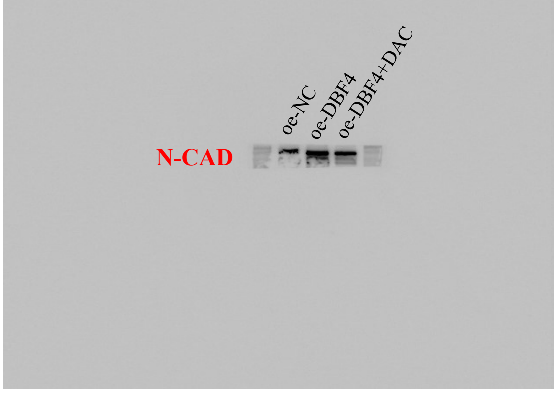

Figure 6E

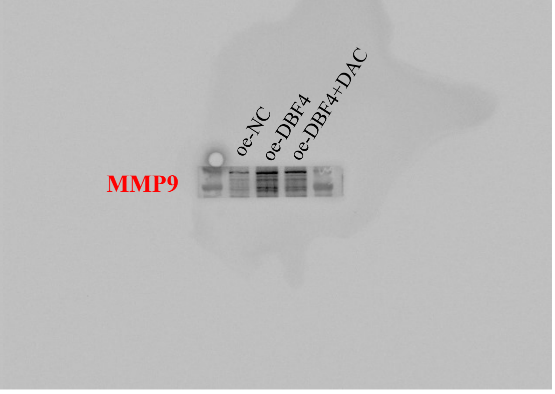

Figure 6E

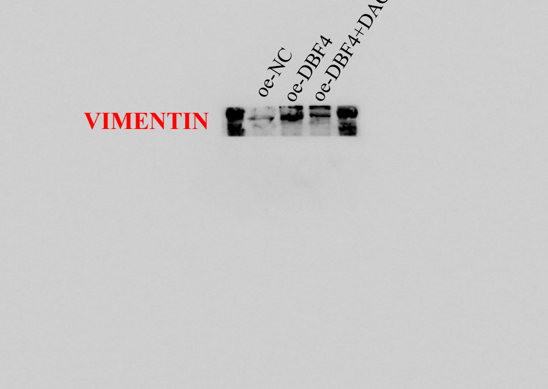

Figure 6E

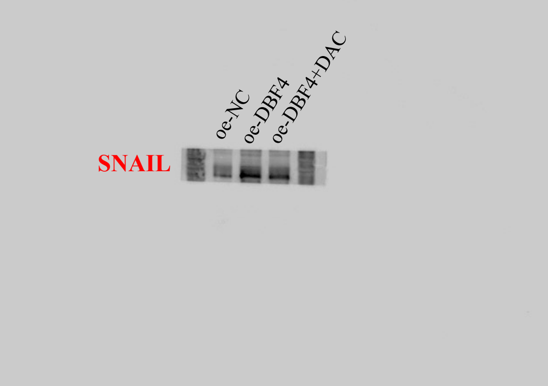

Figure 6E

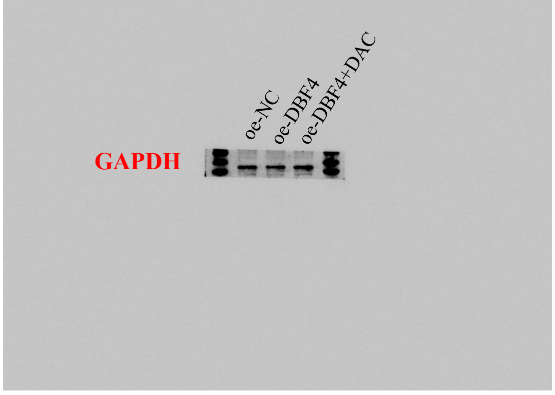

Figure S4

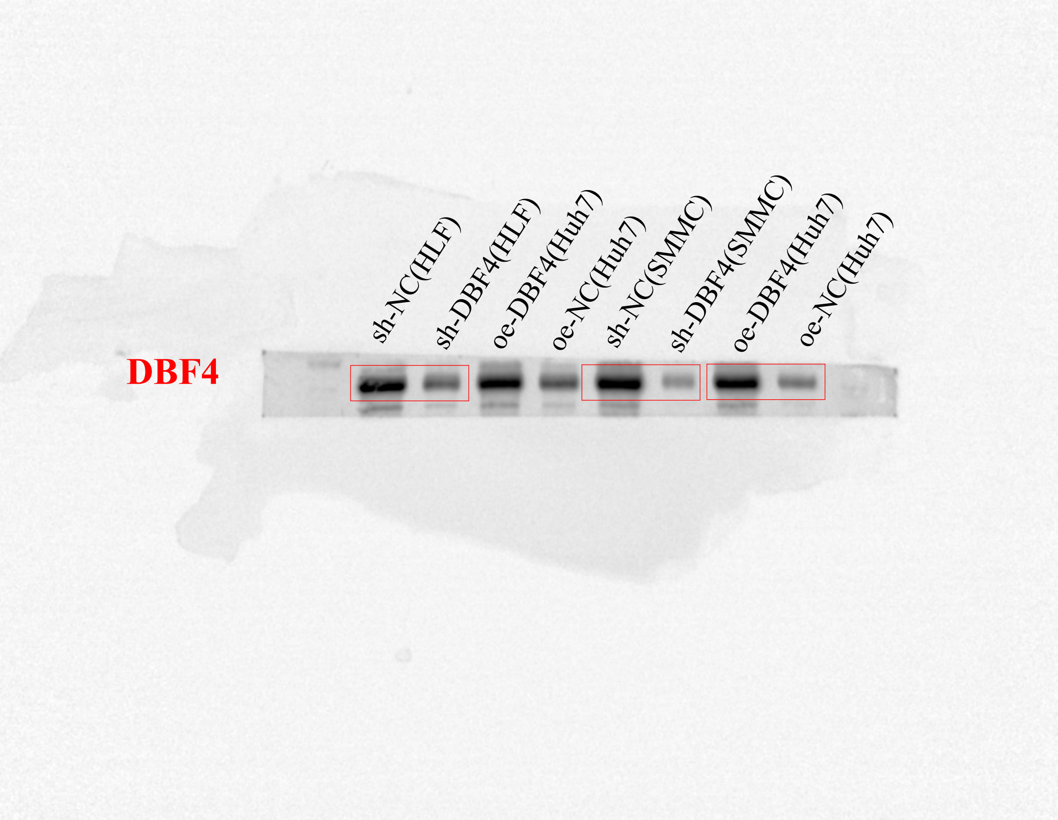

Figure S4

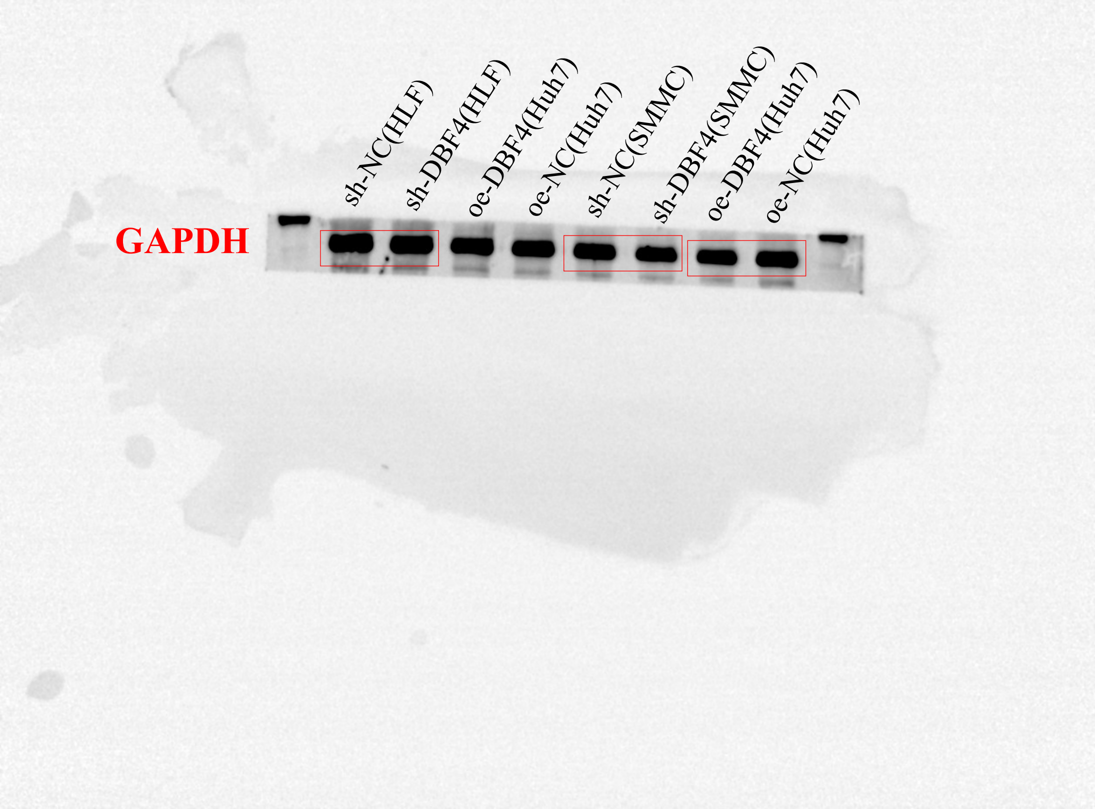

Figure S5

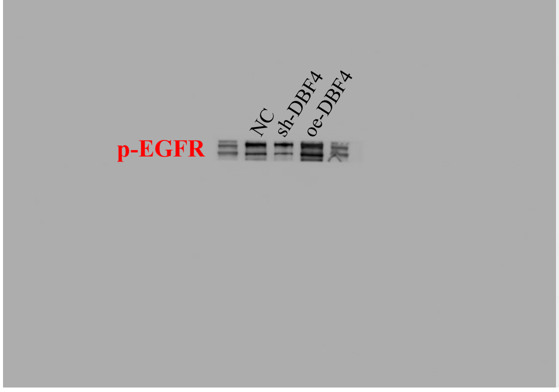

Figure S5

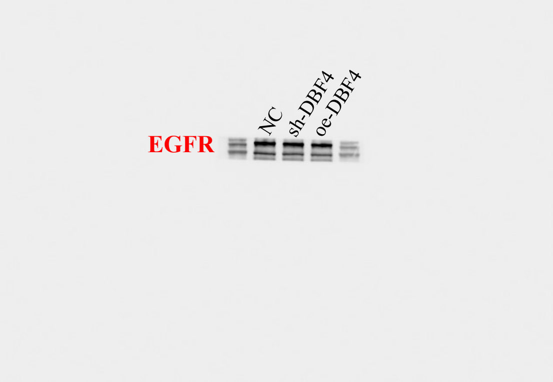

Figure S5

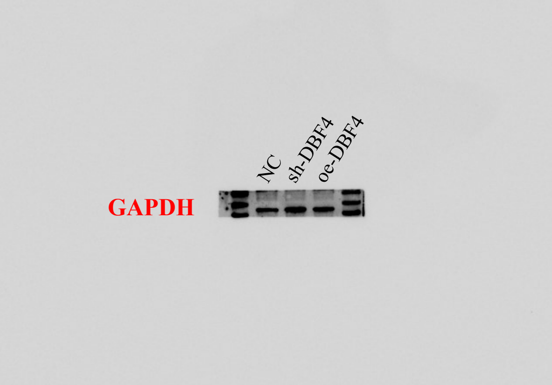

Supplement: Supplementary file 1 — Supplementary Information 1. [file 41598_2024_60342_MOESM1_ESM.pdf]
