# Supplementary material for: The prognostic significance and potential mechanism of DBF4 zinc finger in hepatocellular carcinoma
Source: Sci Rep. 2024 May 9;14:10662. doi: 10.1038/s41598-024-60342-w (PMC11082141; doi:10.1038/s41598-024-60342-w)

Table S1 The association between DBF4 expression and clinicopathological features in LIHC

**Clinical variables**	**No. of patients**	**DBF4 expression level**		***P* value**
	**n=40**	**Low(n=20)**	**High(n=20)**	
Gender				
Male	29	15	14	0.723
Female	11	5	6	
Age (years)				
<60	31	16	15	0.705
≥60	9	4	5	
HBsAg				
Positive	36	19	17	0.598
Negative	4	1	3	
AFP (ng/ml)				
≥400	24	13	11	0.519
<400	16	7	9	
Liver cirrhosis				
Yes	28	13	15	0.490
No	12	7	5	
Child-Pugh Class				
B	7	1	6	0.096
A	33	19	14	
Tumor size (cm)				
≥5cm	27	9	18	0.002
<5cm	13	11	2	
Vascular invasion				
Yes	8	1	7	0.048
NO	32	19	13	
Tumor differentiation				
Poor	17	8	9	0.749
Well	23	12	11	
TNM stage				
III-IV	11	2	9	0.034
I-II	29	18	11	

Figure S1 expression of DBF4 gene. mRNA expression of the DBF4 gene in various cancers and its corresponding normal tissues using the TCGA data (A) and Oncomine database(B). Analysis of pairwise differences in TCGA datasets (C). ($^{**}p < 0.01$, $^{***}p < 0.001$)

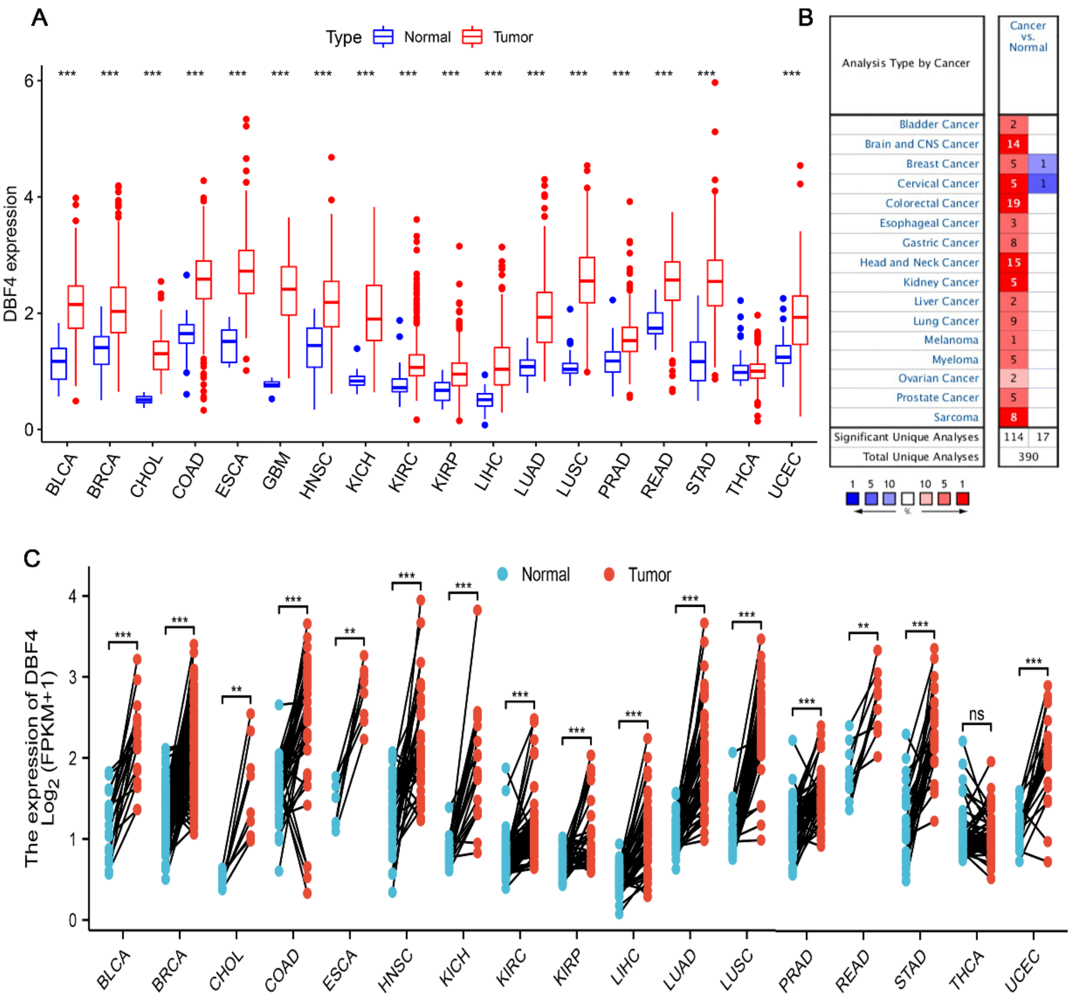

Figure S2 Prognostic potential of DBF4 in cancers. Relationship between DBF4 expression and overall survival (A), disease-specific survival (B) using univariate Cox regression and Kaplan-Meier survival methods.

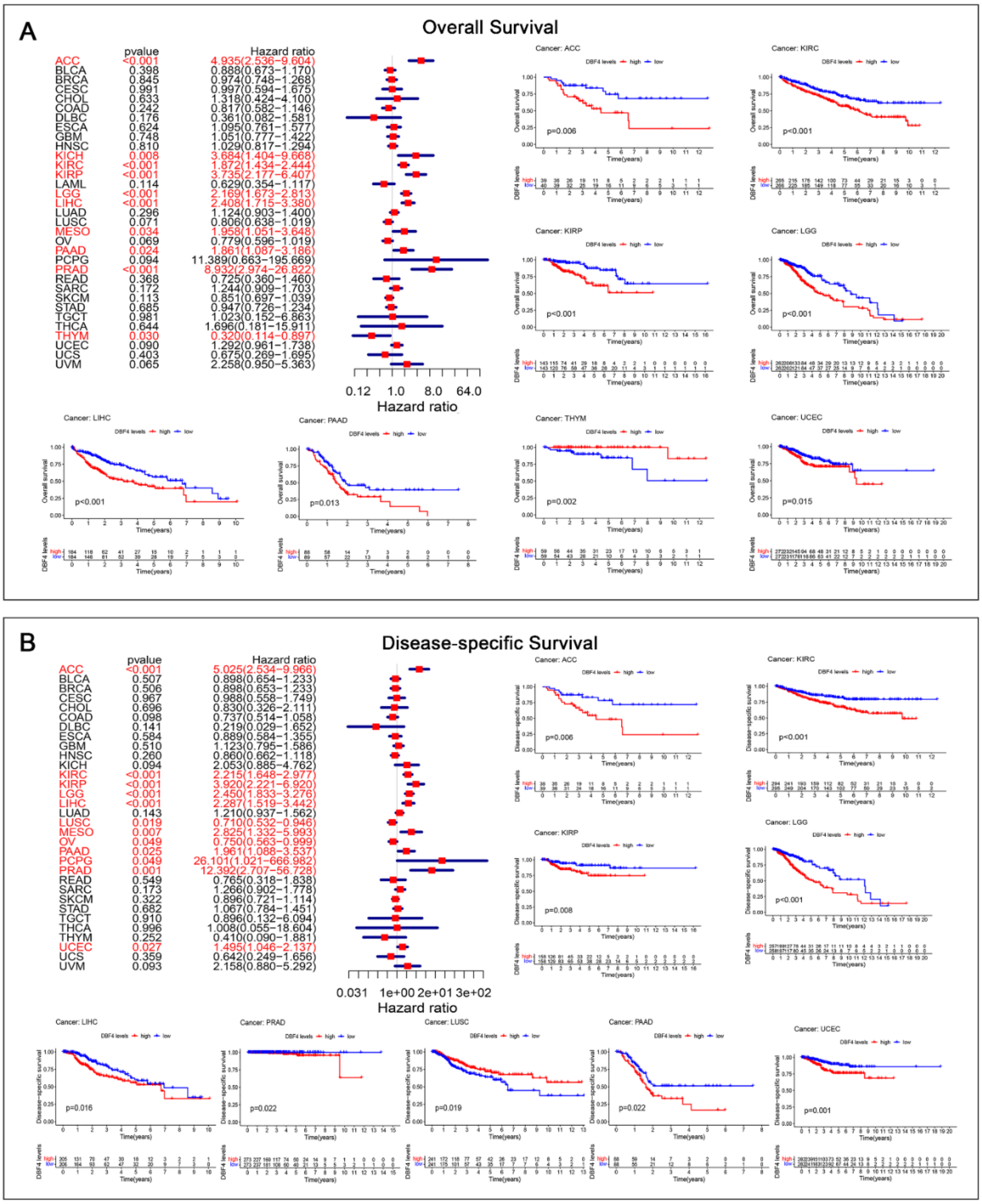

Figure S3 Prognostic potential of DBF4 in cancers. Relationship between DBF4 expression and progression-free interval (A), disease-free interval (B), and relapse-free survival (C) using univariate Cox regression and Kaplan-Meier survival methods.

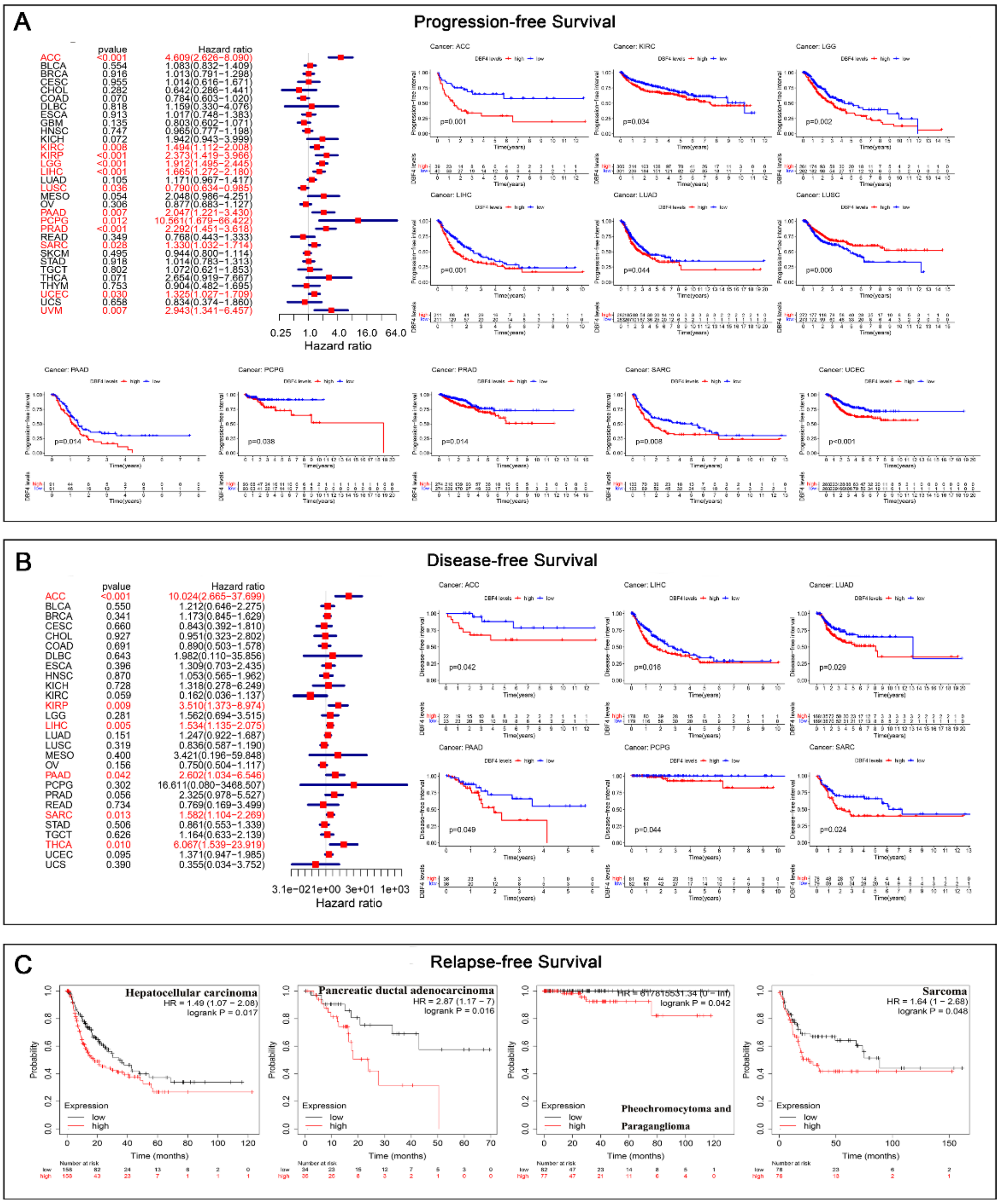

Figure S4 The western blot method was used to assess the expression of DBF4 after knockdown or overexpression with lentivirus-mediated transfection ($^{****}p < 0.0001$).

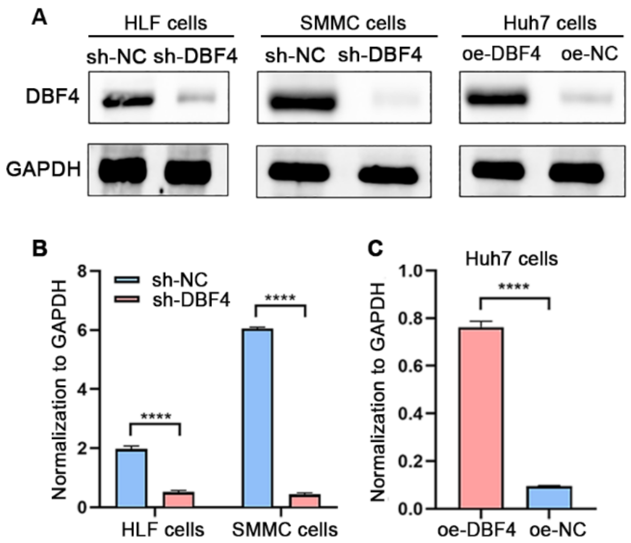

Figure S5 The western blot method was used to assess the expression of p-EGRF after knockdown or overexpression with lentivirus-mediated transfection ($^{**}p < 0.01$, $^{***}p < 0.001$).

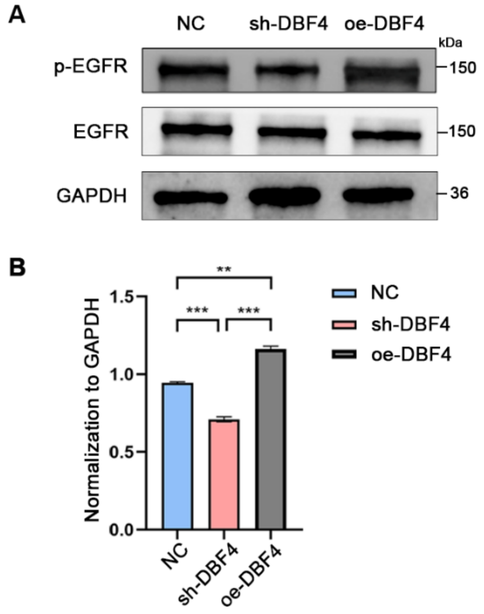

Supplement: Supplementary file 2 — Supplementary Information 2. [file 41598_2024_60342_MOESM2_ESM.pdf]
